# The Conserved LncRNA DIO3OS Restricts Hepatocellular Carcinoma Stemness by Interfering with NONO‐Mediated Nuclear Export of ZEB1 mRNA

**DOI:** 10.1002/advs.202301983

**Published:** 2023-06-04

**Authors:** Ya‐Rui Hou, Li‐Ting Diao, Yan‐Xia Hu, Qian‐Qian Zhang, Guo Lv, Shuang Tao, Wan‐Yi Xu, Shu‐Juan Xie, Qi Zhang, Zhen‐Dong Xiao

**Affiliations:** ^1^ Biotherapy Center The Third Affiliated Hospital Sun Yat‐sen University Guangzhou 510630 P. R. China; ^2^ School of Life Sciences and Biopharmaceutics Guangdong Pharmaceutical University Guangzhou 510006 P. R. China; ^3^ Guangdong Key Laboratory of Liver Disease Research The Third Affiliated Hospital Sun Yat‐sen University Guangzhou 510630 P. R. China; ^4^ Institute of Vaccine The Third Affiliated Hospital Sun Yat‐sen University Guangzhou 510630 P. R. China

**Keywords:** cancer stemness, hepatocellular carcinoma, lncRNA DIO3OS, mRNA subcellular distribution, NONO, ZEB1

## Abstract

Hepatocellular carcinoma (HCC) is an aggressive and fatal disease caused by a subset of cancer stem cells (CSCs). It is estimated that there are approximately 100 000 long noncoding RNAs (lncRNAs) in humans. However, the mechanisms by which lncRNAs affect tumor stemness remain poorly understood. In the present study, it is found that DIO3OS is a conserved lncRNA that is generally downregulated in multiple cancers, including HCC, and its low expression correlates with poor clinical outcomes in HCC. In in vitro cancer cell lines and an in vivo spontaneous HCC mouse model, DIO3OS markedly represses tumor development via its suppressive role in CSCs through downregulation of zinc finger E‐box binding homeobox 1 (ZEB1). Interestingly, DIO3OS represses ZEB1 post‐transcriptionally without affecting its mRNA levels. Subsequent experiments show that DIO3OS interacts with the NONO protein and restricts NONO‐mediated nuclear export of ZEB1 mRNA. Overall, these findings demonstrate that the DIO3OS‐NONO‐ZEB1 axis restricts HCC development and offers a valuable candidate for CSC‐targeted therapeutics for HCC.

## Graphical abstract

This study reveals a crucial role of the conserved lncRNA, DIO3OS, in inhibiting the progression of hepatocellular carcinoma (HCC). DIO3OS binds to NONO protein in the nucleus, which reduces the ability of NONO to promote the nuclear export of ZEB1 mRNA. By regulating the cellular distribution of ZEB1 mRNAs, DIO3OS decreases translation of ZEB1 and subsequently suppresses HCC stemness.

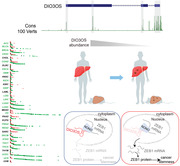

## 1 Introduction

As a leading health challenge worldwide, the morbidity and mortality of hepatocellular carcinoma (HCC) are stubbornly high, although many therapeutic advances have been made.^[^ ^1^, ^2^ ^]^ Numerous studies have proposed that a small cohort of cells called cancer stem cells (CSCs) within tumors are responsible for cancer initiation, propagation, therapy resistance and recurrence.^[^ ^3^, ^4^ ^]^ When cancer exacerbates, CSCs within tumors tend to aberrantly upregulate several stemness‐associated factors, including zinc finger E‐box binding homeobox 1 (ZEB1), to promote therapeutic resistance.^[^ ^4^, ^5^, ^6^, ^7^ ^]^ Thus, identifying the mechanisms of liver CSC would undoubtedly promote not only an in‐depth understanding of the pathogenesis of HCC but also the development of more effective targeted therapies.

Long noncoding RNAs (lncRNAs) are important regulators of gene expression and outnumber protein‐coding genes in the human genome. However, most lncRNAs undergo weak selective pressures, and it has been argued that most lncRNAs may be nonfunctional.^[^ ^8^, ^9^ ^]^ Although conservation is not a general phenomenon,^[^ ^10^ ^]^ in vitro and in vivo studies have shown that several conserved lncRNAs play crucial roles in various physiological and pathological processes.^[^ ^11^, ^12^, ^13^, ^14^, ^15^, ^16^ ^]^ Thus, conservation appears to be highly suggestive of functional importance, and it is of great interest to explore the functions of conserved lncRNAs in various biological processes, especially in cancer, to identify effective targets for therapy.

Mirroring the functional diversity of lncRNAs, their mechanism of action is extremely complex and involves distinct lncRNA‐molecule interactions.^[^ ^17^ ^]^ Some lncRNAs interact with chromatin and recruit chromatin modifiers to activate or suppress transcription.^[^ ^18^ ^]^ Some lncRNAs containing protein interaction domains, act as scaffolds for proteins required to form complexes and function properly.^[^ ^19^, ^20^ ^]^ Some other lncRNAs may interact with RNAs; for example, many lncRNAs bearing microRNA complementary sites can regulate gene expression as sponges of microRNAs, thereby regulating the translation of mRNAs.^[^ ^21^ ^]^ Nonetheless, novel molecular mechanisms remain elusive and more studies are needed to comprehensively elucidate the biology of lncRNAs to facilitate the development of lncRNA‐centered therapeutic strategies.

Here, we identified a conserved lncRNA, DIO3OS, that is downregulated in almost all cancers, including HCC. Subsequently, its conserved role in suppressing HCC stemness was revealed using in vitro HCC cell lines and in vivo mouse models of HCC. Interestingly, we found that DIO3OS binds to the NONO protein and diminishes the effect of NONO on promoting ZEB1 mRNA nuclear export, thereby lessening ZEB1 protein translation and suppressing stemness features. Overall, our findings revealed that DIO3OS, a regulator of ZEB1, suppressed the stemness of HCC and may serve as a potential target for CSC‐targeted therapies.

## 2 Results

### 2.1 DIO3OS, a Conserved lncRNA, Is Frequently Downregulated and Associated with Patient Prognosis in HCC

**Figure 1:**
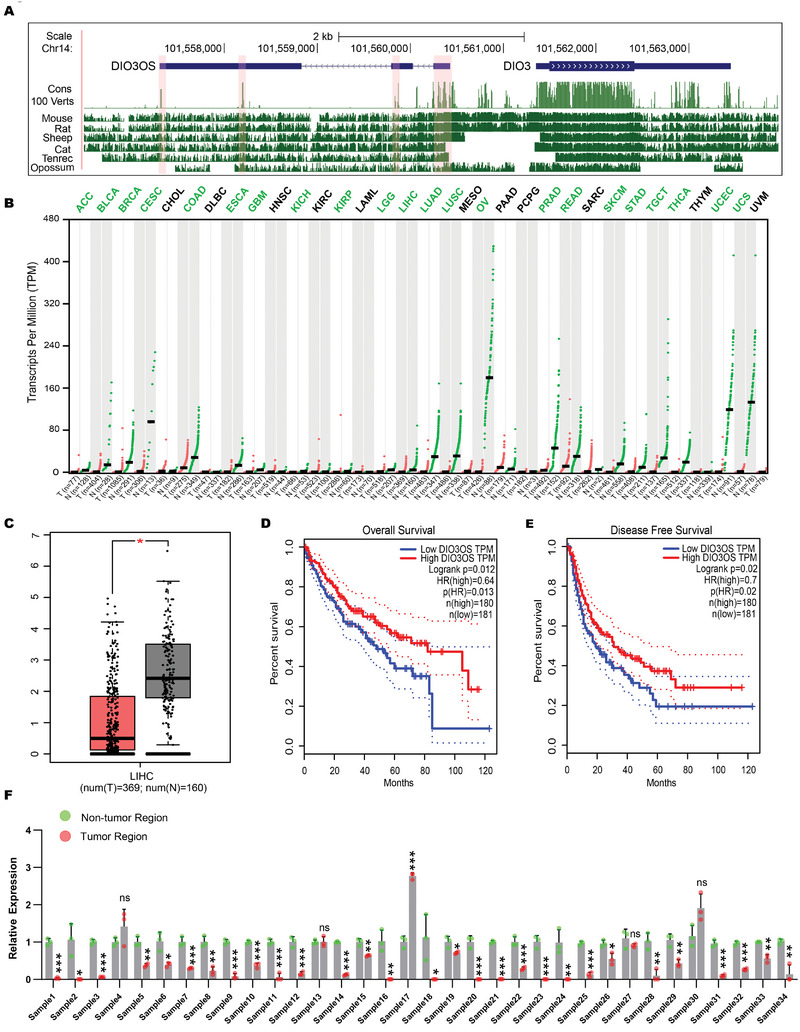
DIO3OS is a highly conserved lncRNA that is downregulated in cancers. A) Genome browser representation of phastCons and basewise conservation tracks at the DIO3OS locus. Cons 100 Verts track shows conservation in 100 vertebrate species measured by phastCons. Basewise conservation between human and other species are displayed in tracks with corresponding species names. Highly conserved blocks along DIO3OS are highlighted by pink shaded boxes. B) Expression profile of DIO3OS from TCGA and GTEx database analyzed by GEPIA (http://gepia.cancer‐pku.cn/). The gene expression profile across all tumor samples and normal tissues is shown in dot plot. Expressions from normal and tumor tissues are shown as green and red dots respectively. Abbreviations of cancers with DIO3OS statistically significantly downregulated are marked in green. C) Exp ression profile of DIO3OS in HCC tissues (*n* = 369 independent specimens) and noncancerous liver tissues (*n* = 160 independent specimens) analyzed by GEPIA. D,E) Kaplan–Meier analysis of the correlations between DIO3OS level and overall survival or disease‐free survival (*n* (high) = 180, *n* (low) = 181) analyzed by GEPIA. F) Real‐time RT‒PCR profile of DIO3OS in 34 pairs HCC clinical samples. Data (F), data are shown as mean ± s.d. of three independent experiments and were analyzed using two‐tailed Student's *t*‐test. ∗*p* < 0.05, ∗∗*p* < 0.01, ∗∗∗*p* < 0.001, and ns, not significant.

Unlike many lncRNAs that are present only in specific species, DIO3OS is highly conserved among species (**Figure** **1**A). Interestingly, we found that DIO3OS was generally downregulated in cancers (Figure 1B; Figure S1A–H, Supporting Information). Occasionally, the expression levels of DIO3OS are associated with patient survival in several cancers (Figure S1I–L, Supporting Information). DIO3OS expression was markedly reduced in HCC (Figure 1C). The log‐rank test showed that patients with HCC with higher DIO3OS had better overall survival and less frequently developed recurrence (Figure 1D,E). To confirm that DIO3OS is downregulated in HCC, we profiled its expression in 34 pairs of clinical samples from HCC tumor tissues and matched adjacent non‐tumor tissues using real‐time RT‐PCR. The levels of DIO3OS were significantly downregulated in 30 samples compared to those in matched non‐tumor tissues (Figure 1F). Given that DIO3OS is highly conserved and is generally downregulated in cancers, it may have potential antitumor functions, particularly in HCC.

### 2.2 Overexpression of DIO3OS Attenuated HCC Stemness Both In Vitro and In Vivo

**Figure 2:**
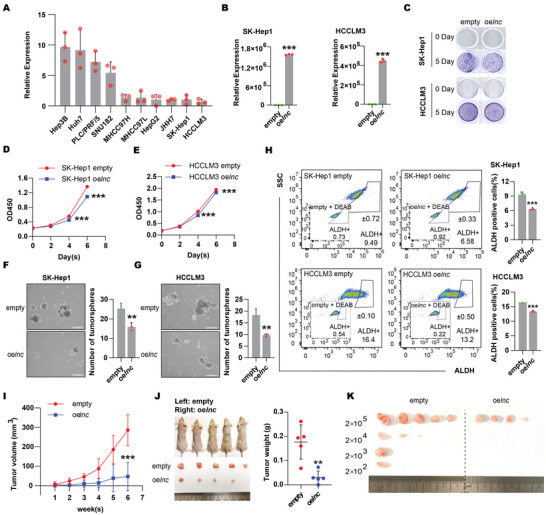
Overexpression of DIO3OS inhibited growth, invasion and stemness of HCC cells. A) Expression profile of DIO3OS in 10 HCC cell lines were detected by real‐time RT‒PCR. B) SK‐Hep1 or HCCLM3 cells stably expressing empty control (empty) or *DIO3OS* (oe*lnc*) were subjected to real‐time RT‒PCR for the measurement of DIO3OS RNA levels. C) Representative images obtained from colony forming assays for SK‐Hep1 or HCCLM3 cells stably expressing empty control or DIO3OS. D,E) Cell proliferations were also measured by Cell Counting Kit‐8 (CCK‐8) for SK‐Hep1 or HCCLM3 stably transfecting with lentivirus encoding empty control sequence or DIO3OS cells. Sphere forming assays for F) SK‐Hep1 or G) HCCLM3 cells stably transfecting with lentivirus encoding empty control sequence or DIO3OS. Representative images from experiments are shown, the numbers of spheroids are quantified in bar graphs. Scale bars, 500 µm. H) Percentages of ALDH+ population of SK‐Hep1 or HCCLM3 *DIO3OS*‐overexpressing cells or their empty control cells analyzed by flow cytometry. Representative images of ALDH+ populations are shown, and bar graphs show quantification from three independent experiments. I,J) SK‐Hep1 stably overexpressing DIO3OS cells and their control empty cells were subcutaneously injected into the left or the right flanks of mice. Tumor volumes were measured weekly one week after injection. At 6 weeks, the mice were sacrificed and tumors were isolated. Images of gross morphology from subcutaneous tumors are displayed, and tumor weight is shown in J) scatter plot. *n* = 5 BALB/C‐nu mice. K) Limiting dilution tumorigenicity analysis of DIO3OS‐overexpressing SK‐Hep1 cells. Different number (2 × 10^5^, 2 × 10^4^, 2 × 10^3^, and 2 × 10^2^) of control and DIO3OS‐overexpressing SK‐Hep1 cells were subcutaneously injected into mice. Photograph shows xenografted tumors from different dilution groups at 7 weeks. *n* = 5 BALB/C‐nu mice for each group. Data (A, B and D–H) are shown as mean ± s.d. of three independent experiments, data from (B), (F–H) were analyzed using two‐tailed Student's *t*‐test; data from (D, E) were analyzed two‐way ANOVA with Tukey's multiple comparisons test. For (I, J), data are shown as mean ± s.d. and were analyzed using two‐way ANOVA with Tukey's multiple comparisons test (I) or two‐tailed Student's *t*‐test (J). ∗∗*p* < 0.01, ∗∗∗*p* < 0.001.

To study the function of DIO3OS in vitro, we first profiled its expression in a panel of HCC cell lines. SK‐Hep1 and HCCLM3 cells, which had lower expression levels than other HCC cell lines (**Figure** **2**A), were selected to assay the gain‐of‐function of DIO3OS (Figure 2B). Similar to a previous study,^[^ ^22^ ^]^ aberrant DIO3OS accumulation mildly reduced cell proliferation, as detected by crystal violet staining and Cell Counting Kit‐8 (CCK‐8) assays (Figure 2C–E). However, we found that DIO3OS strongly repressed the invasive capacity but slightly decreased the migratory ability of SK‐Hep1 cells, as detected by Transwell invasion and migration assays (Figure S2A,B, Supporting Information). Furthermore, ectopic DIO3OS overexpression markedly inhibited the stemness of HCC cells, as determined by tumor sphere formation and ALDEFLUOR assays. We found that DIO3OS overexpression reduced both the number and size of SK‐Hep1 and HCCLM3 tumorspheres (Figure 2F,G). Consistent with the tumorsphere assay results, a lower percentage of ALDH‐positive cells was detected in the ALDEFLUOR assay in DIO3OS‐overexpressing HCC cells (Figure 2H). These data demonstrate that DIO3OS suppresses the development of an aggressive phenotype, especially the stemness, of HCC in vitro.

Subcutaneous transplantation and limiting dilution assays were performed in vivo in nude mice. Consistent with the in vitro results, subcutaneous xenograft models showed that DIO3OS overexpression significantly retarded the growth rate and reduced the tumor weight of SK‐Hep1 cells (Figure 2I,J). In addition, overexpression of DIO3OS led to a significantly reduced xenograft incidence of SK‐Hep1 cells in the subcutaneous transplantation of serially decreasing numbers of tumor cells in nude mice, suggesting an in vivo repressing role of DIO3OS in HCC stemness (Figure 2K; Figure S2C–E, Supporting Information). Taken together, our results demonstrate that DIO3OS attenuates the stemness of HCC cells both in vitro and in vivo.

### 2.3 Depletion of DIO3OS Promoted HCC Stemness In Vitro and In Vivo

**Figure 3:**
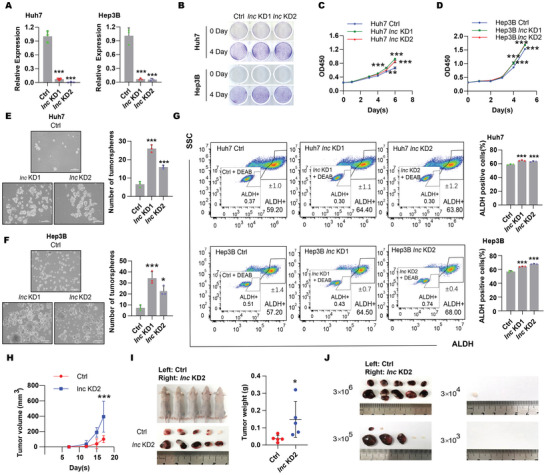
Transcriptional inhibition of DIO3OS promoted HCC stemness both in vitro and in vivo. A) The RNA levels of DIO3OS were examined by real‐time RT‒PCR in Huh7 or Hep3B cells stably expressing dCas9‐Krab‐control sgRNA (Ctrl) or dCas9‐Krab‐DIO3OS sgRNA (*lnc* KD; targeting downstream of DIO3OS transcription start site). B) Representative images from colony forming assays of Huh7 or Hep3B cells stably expressing control sgRNA or *DIO3OS*‐targeting sgRNAs. C,)Cell proliferations were assessed by Cell Counting Kit‐8 in C) Huh7 and D) Hep3B cells when DIO3OS was stably knocked down compared to their control cells, respectively. E) Huh7 and F) Hep3B cells infected with lentivirus encoding control sgRNA or DIO3OS‐targeting sgRNAs were subjected to sphere forming assays. Representative images are shown, the numbers of spheroids were quantified and shown in bar graphs. Scale bars, 200 µm. G) Flow cytometry analysis of ALDH+ population of Huh7 or Hep3B cells with stably expressing control sgRNAs or DIO3OS‐targeting sgRNAs. Representative images are shown, and bar graphs show quantification from the three independent experiments. H,I) Huh7 DIO3OS‐depleted cells and control cells were subcutaneously injected into the left or the right flanks of mice. Tumor volumes were measured every 3 days one week after injection and shown as growth curve (H). At 17 days, mice were sacrificed and tumors were isolated. Images of gross morphology from subcutaneous tumors are displayed, and tumor weight is shown in I) scatter plot. *n* = 5 BALB/C‐nu mice. J) Limiting dilution tumorigenicity analysis of DIO3OS‐depleted Huh7 cells. Different number (3 × 10^6^, 3 × 10^5^, 3 × 10^4^, and 3 × 10^3^) of Huh7 control cells and DIO3OS knocked down cells were subcutaneously injected into the left or the right flanks of mice, respectively. At 46 days, all mice have been sacrificed and tumors were isolated. Xenografted tumors from different dilution groups were shown. *n* = 5 BALB/C‐nu mice for each group. Data (A, C–G) are shown as mean ± s.d. of three independent experiments, data from (A, E–G) were analyzed using one‐way ANOVA with Tukey's multiple comparisons test; data from (C, D) were analyzed two‐way ANOVA with Tukey's multiple comparisons test. For (H, I), data are shown as mean ± s. d. and were analyzed using two‐way ANOVA with Tukey's multiple comparisons test (H) or two‐tailed Student's *t*‐test (I). ∗*p* < 0.05, ∗∗ *p* < 0.01, ∗∗∗*p* < 0.001.

To further assess the importance of DIO3OS, we used a loss‐of‐function strategy to study its function. Higher DIO3OS expression was observed in Hep3B and Huh7 cells (Figure 2A). We stably knocked down DIO3OS in Huh7 and Hep3B cells using CRISPR interference (**Figure** **3**A). These results were consistent with those of the gain‐of‐function study; knockdown of DIO3OS mildly enhanced the growth and migration of HCC cells (Figure 3B–D; Figure S3C,D, Supporting Information). However, knockdown of DIO3OS not only greatly boosted the number of invading cells in the Transwell invasion assay (Figure S3A,B, Supporting Information), but also notably augmented the number of tumorspheres and ALDH‐positive cells in the sphere forming and ALDEFLUOR assays, respectively (Figure 3E,G). To further examine the effects of DIO3OS depletion in vivo, Huh7 cells with stable DIO3OS knockdown were injected into nude mice for subcutaneous transplantation and limiting dilution assays. Depletion of DIO3OS significantly accelerated tumor growth in vivo (Figure 3H,I) and strongly increased the tumorigenesis frequency of Huh7 cells after subcutaneous injection of a series of cell dilutions (Figure 3J; Figure S3E,F, Supporting Information). Taken together, our results suggest that DIO3OS depletion promotes the stemness of HCC cells both in vitro and in vivo.

### 2.4 DIO3OS Modulated HCC Stemness through Regulation of ZEB1 mRNA Subcellular Location

**Figure 4:**
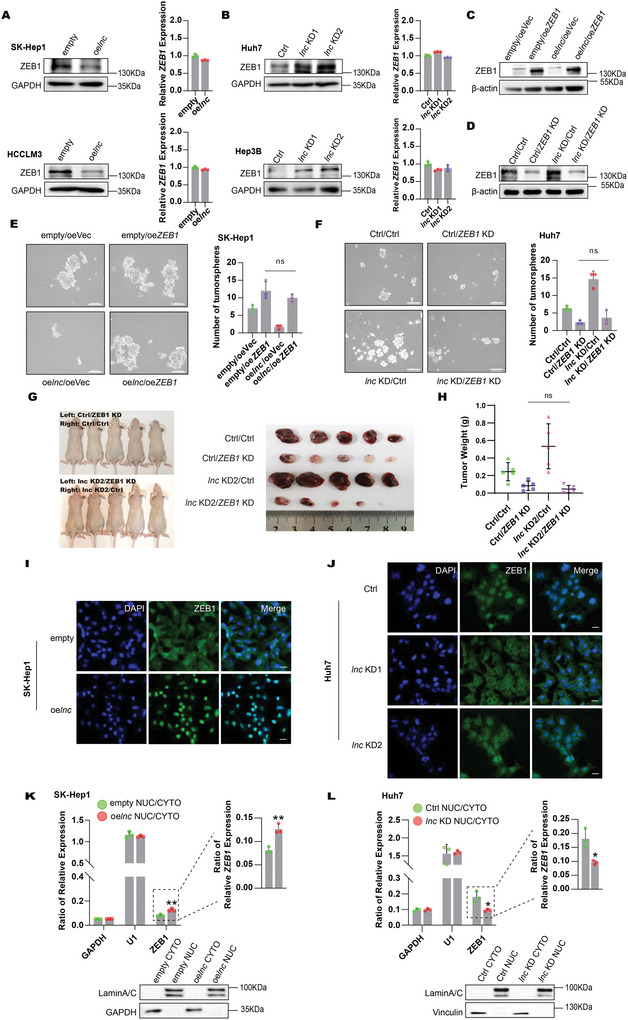
DIO3OS repressed HCC stemness by modulating ZEB1 mRNA subcellular location. A,B) DIO3OS was overexpressed in SK‐Hep1 and HCCLM3 cells and was knocked down in Huh7 and Hep3B cells. Protein and mRNA levels of ZEB1 were detected by western blotting and real‐time RT‒PCR, respectively. GAPDH was used as loading control for western blotting. C,D) SK‐Hep1 cells with stable expression of empty control (empty) or *DIO3OS* (oe*lnc*) were infected with empty (oeVec) or *ZEB1* (oe*ZEB1*) lentivirus. Huh7 cells with stable expression of control vector (Ctrl) or knocked‐down *DIO3OS* (*lnc* KD) were infected with control (Ctrl) or *ZEB1* knocked‐down lentivirus (*ZEB1* KD). Protein levels of ZEB1 were detected by western blotting, *β*‐actin was used as loading control. E,F) Sphere forming assays of SK‐Hep1 cells when DIO3OS and ZEB1 were both overexpressed and Huh7 cells when DIO3OS and ZEB1 were both knocked down. Representative images from experiments are shown, the numbers of spheroids are quantified and shown in bar graphs. Scale bars, 200 µm. G,H) DIO3OS‐depleted Huh7 cells with or without ZEB1 knocking down were subcutaneously injected into the left or the right flanks of mice. At 17 days, mice were sacrificed and tumors were isolated. G) Images of gross morphology from subcutaneous tumors are displayed, and H) tumor weight is shown in scatter plot. *n* = 5 BALB/C‐nu mice. I,J) RNA FISH detection using probes targeting ZEB1 in SK‐Hep1 or Huh7 cells transfecting with the indicated lentivirus. ZEB1 was stained in Green. DNA was stained with DAPI (blue). Scale bars, 20 µm. K,L) Cytoplasmic and nuclear fractionation analysis of SK‐Hep1 DIO3OS‐overexpressing cells and Huh7 DIO3OS‐depleted cells. Nuclear and cytosolic extracts were subjected to western blotting and real‐time RT‒PCR. GAPDH and U1 served as markers of cytoplasm and nucleus in real‐time RT‒PCR, respectively. GAPDH/Vinculin and Lamin A/C served as markers of cytoplasm and nucleus in western blotting, respectively. Data (A, B, E, F, K, and L) are shown as mean ± s.d. of three independent experiments, data from (E) and (F) were analyzed using one‐way ANOVA with Tukey's multiple comparisons test; (K) and (L) were analyzed using two‐tailed Student's *t*‐test. For (H), data are shown as mean ± s. d. and were analyzed using one‐way ANOVA with Tukey's multiple comparisons test. ∗*p* < 0.05, ∗∗*p* < 0.01, and ns, not significant.

Mounting evidence indicates the crucial role of several master stemness‐associated transcription factors in tumor stemness.^[^ ^4^, ^7^ ^]^ Among the factors tested, the protein levels of ZEB1 strikingly increased in different DIO3OS‐depleted HCC cells (**Figure** **4**B; Figure S4A,B, Supporting Information) and significantly decreased in DIO3OS‐overexpressing HCC cells (Figure 4A), whereas ZEB1 mRNA levels remained unchanged (Figure 4A,B). Additionally, of the 34 HCC clinical samples, 23 showed DIO3OS downregulation and ZEB1 upregulation (Figure S4C, Supporting Information). There was a significant negative correlation between DIO3OS and ZEB1 levels (Figure S4D, Supporting Information). In addition, ZEB1 protein expression was examined in 12 HCC samples by immunohistochemical staining, and a strong negative correlation between DIO3OS and ZEB1 was observed (Figure S4E, Supporting Information). These results further indicate that DIO3OS represses ZEB1 protein expression via post‐transcriptional regulation, and that ZEB1 may be a key downstream target of DIO3OS. To explore whether the alteration in stemness regulated by DIO3OS was due to ZEB1, rescue assays were performed. Overexpression of ZEB1 in DIO3OS‐overexpressing cells neutralized ZEB1 protein downregulation (Figure 4C), whereas ZEB1 knockdown in DIO3OS‐depleted cells minimized ZEB1 protein upregulation (Figure 4D). Consistently, overexpression or knockdown of ZEB1 restored the effects of overexpression or knocking down DIO3OS on HCC stemness in the tumorsphere‐forming assays (Figure 4E,F). Consistently, in the xenograft tumor model, ZEB1 knockdown significantly neutralized DIO3OS deficiency‐induced tumor growth (Figure 4G,H). Thus, DIO3OS modulates HCC stemness via the DIO3OS‐ZEB1 axis.

Given that DIO3OS modulated the protein level of ZEB1 without altering its mRNA level, we reasoned that DIO3OS may regulate the degradation of ZEB1 protein or the subcellular location of ZEB1 mRNA. Notably, DIO3OS did not decrease ZEB1 stability (Figure S5A,B, Supporting Information). Instead, we found that DIO3OS affected the subcellular location of ZEB1 mRNAs. Using RNA fluorescence in situ hybridization (RNA FISH), we found that ZEB1 mRNAs predominantly reside in the nucleoplasm when DIO3OS is overexpressed (Figure 4I). However, DIO3OS deficiency promoted ZEB1 mRNA translocation out of the nucleus (Figure 4J). Furthermore, we assessed the localization of ZEB1 mRNAs by cell fractionation analysis. DIO3OS accumulation resulted in greater ZEB1 mRNA retention in the nucleus, whereas DIO3OS depletion resulted in a higher proportion of ZEB1 mRNA in the cytoplasm (Figure 4K,L). In summary, our data indicate that ZEB1 is an important downstream target of DIO3OS and that DIO3OS monitors ZEB1 by modulating the cellular distribution of its mRNA.

### 2.5 DIO3OS Suppressed NONO‐Mediated ZEB1 mRNA Nuclear Export

**Figure 5:**
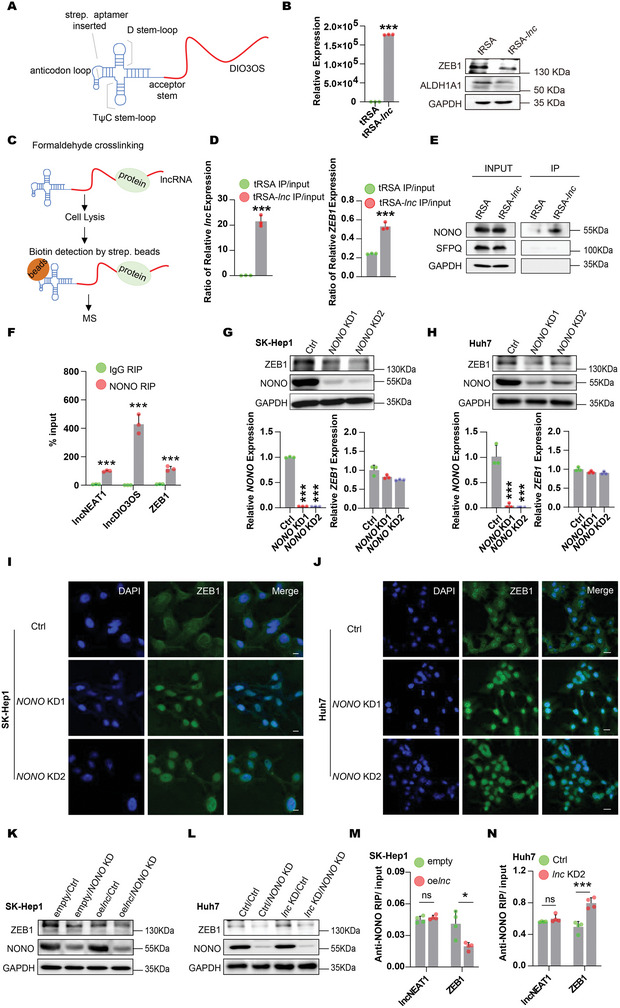
DIO3OS‐NONO‐ZEB1 mRNA axis played a crucially regulatory role in HCC stemness. A) Schematic structure of tRSA‐DIO3OS. B) DIO3OS level was detected by real‐time RT‒PCR, and protein levels of ZEB1 and ALDH1A1 were detected by western blotting in SK‐Hep1 tRSA‐DIO3OS overexpressing (tRSA‐*lnc*) and empty control (tRSA) cells. GAPDH was used as loading control in western blotting. C) The flow scheme for tRSA‐DIO3OS pull down assay. D) Enrichments of DIO3OS and ZEB1 RNA levels were examined by real‐time RT‒PCR in tRSA‐DIO3OS pull down. E) NONO, SFPQ, and GAPDH were detected by western blotting in tRSA‐DIO3OS pull down. SFPQ and GAPDH as negative control. F) RIP detection of NEAT1, DIO3OS, and ZEB1 mRNA by using antibody against NONO in Huh7 cells. NEAT1 as positive control. G,H) SK‐Hep1 cells with stable expression of empty control (empty) or *DIO3OS* (oe*lnc*) were infected with control lentivirus (Ctrl) or *NONO* knocked‐down lentivirus (*NONO* KD). Huh7 cells stably expressing control vector (Ctrl) or knocked‐down *DIO3OS* (*lnc* KD) were infected with control lentivirus (Ctrl) or *NONO* knocked‐down lentivirus (*NONO* KD). Western blotting and real‐time RT‒PCR were used to examine the protein and RNA levels of NONO and ZEB1 respectively. GAPDH was used as loading control in western blotting. I,J) RNA FISH detection for ZEB1 mRNA in SK‐Hep1 or Huh7 cells transfecting with the indicated lentivirus. ZEB1 mRNAs were stained in Green. DNA were stained with DAPI (blue). I) Scale bars, 10 µm; J) scale bars, 20 µm. K,L) NONO was knocked down in SK‐Hep1 DIO3OS overexpressing cells. DIO3OS and NONO were both depleted in Huh7 cells. Protein levels of ZEB1 and NONO were confirmed by western blotting, GAPDH was used as loading control. M,N) RIP detection of NEAT1 and ZEB1 RNA using NONO antibody in SK‐Hep1 DIO3OS overexpressing cells and Huh7 DIO3OS knocked down cells. NEAT1 as negative control. Data (B, D, F–H, M, N) are shown as mean ± s.d. of three independent experiments, data from (B, D, F, M, N) were analyzed using two‐tailed Student's *t*‐test; data from (G) and (H) were analyzed using one‐way ANOVA with Tukey's multiple comparisons test. ∗*p* < 0.05, ∗∗∗*p* < 0.001, and ns, not significant.

To further explore the mechanism underlying the nuclear export of ZEB1 mRNA, we identified the protein interactome of DIO3OS. LncRNA‐protein interactome studies are challenging because of the low abundance of lncRNAs and the inefficiency of conventional approaches for in vivo precipitation. To solve this issue, we fused a tRNA‐scaffolded streptavidin aptamer (tRSA) to the 5′ end of DIO3OS, in which DIO3OS may remain in a natural conformation and could be sufficiently immunoprecipitated by streptavidin beads^[^ ^23^, ^24^ ^]^ (**Figure** **5**A). To determine whether tRSA correctly fused with DIO3OS, we measured the expression levels of DIO3OS, ZEB1, and ALDH1A1 in tRSA‐DIO3OS overexpressed SK‐Hep1 cells. Compared with tRSA‐empty cells, DIO3OS RNA levels steadily increased, whereas the protein levels of ZEB1 and ALDH1A1 were significantly downregulated in tRSA‐DIO3OS‐overexpressing SK‐Hep1 cells (Figure 5B), implying that tRSA‐fused DIO3OS functionally modulated stemness.

To identify DIO3OS‐binding proteins in vivo, we stably overexpressed tRSA‐DIO3OS in SK‐Hep1 cells, cross‐linked the cells with formaldehyde, precipitated the DIO3OS‐protein complex with streptavidin beads, and used mass spectrometry (MS) to identify DIO3OS‐interacting proteins (Figure 5C). Among the top proteins identified, NONO was particularly interesting because it is a well‐known regulator of mRNA subcellular localization^[^ ^25^, ^26^ ^]^ (Figure S6 and Table S1, Supporting Information). We also confirmed DIO3OS and ZEB1 RNA levels in tRSA‐DIO3OS immunoprecipitated samples. DIO3OS and ZEB1 mRNA were enriched in the tRSA‐DIO3OS samples (Figure 5D). Western blotting confirmed these mass spectrometry results. The NONO protein was significantly enriched in tRSA‐DIO3OS precipitates compared to the tRSA control, whereas there was no enrichment for GAPDH and SFPQ (Figure 5E). To further validate the interaction between NONO and DIO3OS, we performed RNA immunoprecipitation (RIP) in Huh7 cells. Real‐time RT‐PCR showed obvious enrichment of DIO3OS and NEAT1 (a known NONO‐interacting lncRNA^[^ ^27^, ^28^, ^29^ ^]^) in pull‐downs with an antibody against NONO, confirming the interaction between the NONO protein and DIO3OS RNA (Figure 5F). Interestingly, our RIP assay also revealed a clear interaction between the NONO protein and ZEB1 mRNA (Figure 5F), suggesting that NONO may be a key factor in DIO3OS regulating ZEB1 mRNA subcellular distribution.

To examine the role of NONO in the DIO3OS regulation of ZEB1 mRNA distribution, we first knocked down NONO using CRISPR interference in SK‐Hep1 and Huh7 cells and found that NONO deficiency led to a notable decrease in ZEB1 at the protein level but not at the mRNA level (Figure 5G,H). Next, we investigated whether NONO affects the subcellular localization of ZEB1 mRNA. To explore this, ZEB1 mRNA distribution was monitored by using RNA FISH in SK‐Hep1 and Huh7 cells stably depleted of NONO. ZEB1 mRNAs were arrested in the nucleus in response to NONO deficiency, indicating that NONO facilitates ZEB1 mRNA export to the cytoplasm (Figure 5I,J). We also attenuated NONO expression in DIO3OS‐accumulating or ‐depleted cells. NONO deficiency accelerated the decline in ZEB1 expression in DIO3OS‐overexpressing SK‐Hep1 cells (Figure 5K). In addition, the depletion of NONO neutralized the elevation of ZEB1 in DIO3OS‐depleted Huh7 cells (Figure 5L), suggesting that NONO is required for DIO3OS to modulate ZEB1 mRNA distribution. Additionally, we performed RIP to measure the ability of NONO to capture ZEB1 mRNA in DIO3OS‐accumulating or ‐depleted cells. A decreased interaction between NONO and ZEB1 mRNA was detected when DIO3OS was overexpressed in SK‐Hep1 cells, whereas NEAT1 precipitated by NONO was unaffected by DIO3OS upregulation (Figure 5M). Conversely, we detected an increased affinity between NONO and ZEB1 mRNA when DIO3OS was attenuated in Huh7 cells, and the levels of NONO‐precipitating NEAT1 remained unchanged (Figure 5N), indicating that DIO3OS regulates ZEB1 mRNA nuclear export by repressing the interaction between NONO and ZEB1 mRNAs. Taken together, these data indicate that DIO3OS could modulate ZEB1 mRNA cellular distribution with the help of NONO, leading to nuclear retention of ZEB1 mRNAs.

### 2.6 Dio3os Plays Conserved Suppressor Roles during Murine Hepatic Tumorigenesis

**Figure 6:**
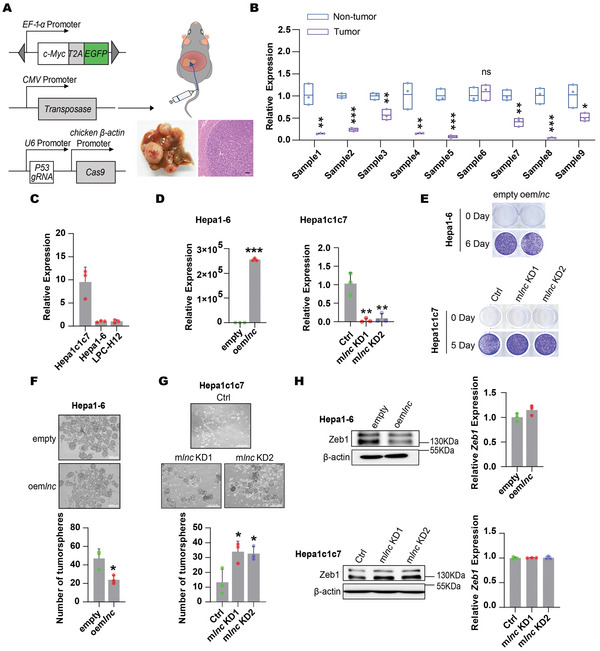
DIO3OS‐ZEB1 axis is conserved in human and mouse. A) c‐Myc, P53 knockout as well as SB‐100 plasmids were mixed in saline and then injected into C57BL/6J mice via tail vein to generate mice primary liver cancer. Photographs show macroscopic and microscopic structure of livers. Scale bars, 100 µm. B) Real‐time RT‒PCR was used to profile Dio3os in 9 murine primary HCC specimens. C) Expression levels of Dio3os in three murine HCC cells were examined by real‐time RT‒PCR. D) The RNA levels of Dio3os were examined in Dio3os overexpressing Hepa1‐6 cells and Dio3os knocked down Hepa1c1c7 cells as well as their control cells by using real‐time RT‐PCR. E) Representative images of colony forming assays for Dio3os‐overexpressing Hepa1‐6 cells and Dio3os‐depleted Hepa1c1c7 cells, respectively. F) Sphere forming assays of Dio3os overexpressing and control Hepa1‐6 cells. Representative images from experiments were shown, the numbers of spheroids were quantified and shown in bar graphs. Scale bars, 200 µm. G) Sphere forming assays of Dio3os depleted and control Hepa1c1c7 cells. Representative images from experiments are shown, the numbers of spheroids are quantified and shown in bar graphs. Scale bars, 500 µm. H) Protein and mRNA levels of Zeb1 were examined in Dio3os overexpressed Hepa1‐6 cells and Dio3os depleted Hepa1c1c7 cells by western blotting and real‐time RT‒PCR, respectively. *β*‐actin was used as loading control in western blotting. Data (C, D, F–H) are shown as mean ± s.d. of three independent experiments, data from (D) (left panel) and (F) were analyzed using two tailed Student's *t*‐test; data from (D) (right panel) and (G) were analyzed using one‐way ANOVA with Tukey's multiple comparisons test. For Figure B, data are shown as mean ± s.d. and was analyzed using two‐tailed Student's *t*‐test. ∗*p* < 0.05, ∗∗*p* < 0.01, ∗∗∗*p* < 0.001, and ns, not significant.

As conserved in mice (Figure 1A), we further assayed the function of Dio3os in mouse HCC. A spontaneous liver cancer model was generated via hydrodynamic tail vein injection (HTVI)^[^ ^30^, ^31^ ^]^ (**Figure** **6**A). In tumors and corresponding non‐tumor tissues from nine murine HCC specimens, Dio3os was significantly downregulated in eight specimens (Figure 6B), suggesting that Dio3os may play a similar suppressive role in mouse HCC tumorigenesis. We first explored the stemness function of Dio3os in vitro by conducting gain‐ and loss‐of‐function assays in murine HCC cell lines. We stably overexpressed Dio3os in Hepa1‐6 cells and knocked down Dio3os in Hepa1c1c7 cells (Figure 6C,D). Overexpression of Dio3os mildly impaired Hepa1‐6 cell proliferation, whereas knockdown of Dio3os slightly increased the propagation of Hepa1c1c7 cells (Figure 6E). Consistent with the results for human DIO3OS, we found that Dio3os expression was markedly associated with HCC stemness. Dio3os accumulation significantly decreased the number and size of tumorspheres formed by Hepa1‐6 cells (Figure 6F), whereas Dio3os deficiency augmented the tumorsphere‐forming capacity of Hepa1c1c7 cells (Figure 6G). To explore whether the DIO3OS‐ZEB1 axis is also conserved, we examined the expression levels of Zeb1 in Dio3os‐altered murine HCC cells. Interestingly, the protein levels of Zeb1 decreased in Dio3os‐ectopically accumulating Hepa1‐6 cells but increased in Dio3os‐depleted Hepa1c1c7 cells, whereas Zeb1 mRNA levels remained unaffected (Figure 6H). Taken together, our in vitro results suggest that Dio3os has a conserved function and a similar underlying mechanism in HCC tumorigenesis.

**Figure 7:**
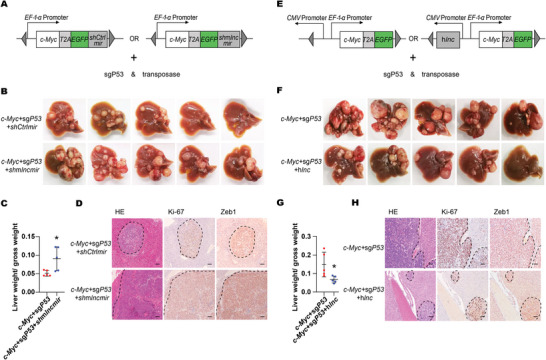
Suppressive effects of Dio3os on murine hepatic tumorigenesis. A) Diagram of miR30 based shRNA construction for Dio3os depletion. Mice were injected with control group vectors (*c‐Myc*+sg*P53*+*shCtrlmir*) and Dio3os knocked down group vectors (*c‐Myc*+sg*P53*+*sh*m*lncmir*) respectively. Mice were then sacrificed at 5 weeks after tail vein injection, *n* = 5 C57BL/6 mice each group. B) Macroscopic graphs of livers from control and Dio3os knocked down mice. C) Relative liver weights of control and Dio3os knocked down mice are shown in scatter plot. D) Microscopic photos of mice livers sections from control and Dio3os knocked down groups. Sections were stained with antibodies against Ki‐67 and ZEB1 by IHC. Scale bars, 100 µm. E) Diagram of human DIO3OS overexpression construction. Mice were divided into control (*c‐Myc*+sg*P53*) group and DIO3OS overexpression (*c‐Myc*+sg*P53*+h*lnc*) group respectively. Mice were then sacrificed at 6 weeks after tail vein injection, *n* = 5 C57BL/6 mice each group. F) Macroscopic graphs of livers from control and DIO3OS overexpressing groups. G) Relative liver weights of control and DIO3OS overexpressing mice are shown by scatter plot. H) Microscopic photos of mice livers sections from control and DIO3OS overexpressing mice. Sections were stained with Ki‐67 and ZEB1 antibodies by IHC. Scale bars, 100 µm. For (C, G), data are shown as mean ± s.d. and were analyzed using two‐tailed Student's *t*‐test. ∗*p* < 0.05.

To validate the tumor‐suppressive role of mouse Dio3os in vivo, we knocked down mouse *Dio3os* using shRNA and scored the effects of Dio3os on HCC induced by *c‐Myc* overexpression and *P53* deficiency (**Figure** **7**A). At necropsy, mice with Dio3os knockdown showed massive, expansile, and multifocal liver tumors (Figure 7B). Additionally, the relative liver weight of the DIO3OS‐depleted group was significantly higher than that of the control group (Figure 7C). Furthermore, positive immunohistochemical staining of both Ki‐67 and Zeb1 in tumor tissues was aggravated when Dio3os was knocked down, suggesting that downregulation of Dio3os promotes HCC in mice (Figure 7D). To further clarify the conserved effects of DIO3OS on HCC stemness, human *DIO3OS* was hydrodynamically injected into mice (Figure 7E). Photographs of liver‐bearing tumors showed that tumor burden and relative liver weight were minimized by DIO3OS overexpression (Figure 7F,G). In addition, immunohistochemical staining for Ki‐67 and Zeb1 in the liver tumors indicated that HCC proliferation and stemness were reduced when DIO3OS was overexpressed (Figure 7H). Furthermore, Zeb1 knockout repressed the promoting effects of Dio3os‐deficiency on hepatocarcinogenesis (Figure S7A,B, Supporting Information), further suggesting that DIO3OS modulated tumorigenesis depends on the conserved DIO3OS‐ZEB1 axis. Collectively, our data demonstrate that DIO3OS exerts conserved suppressive effects on hepatocarcinogenesis.

## 3 Discussion

Approximately 100 000 lncRNAs are encoded in the human genome.^[^ ^32^, ^33^, ^34^ ^]^ However, only a small fraction yielded a clear phenotype both in vitro and in vivo. Here, we identified DIO3OS, a conserved lncRNA, as an important regulator of HCC stemness by modulating ZEB1 nuclear transport using in vitro cell lines, in vivo nude mice, and an HTVI HCC mouse model.

DIO3OS is generally downregulated across cancers, suggesting its potential tumor‐repressing function in multiple tumors. Indeed, DIO3OS was recently reported to play a regulatory role in multiple cancers, including HCC, in a *trans* manner.^[^ ^22^, ^35^, ^36^, ^37^ ^]^ Our results on the DIO3OS‐modulating cancer phenotype, including proliferation and motility, were largely in accordance with those of previous studies, suggesting a robust tumor‐suppressing role of DIO3OS in HCC. Interestingly, compared with proliferation, we observed a more dramatic inhibition of HCC stemness, which was consistent with the identification of ZEB1 as a master downstream effector of DIO3OS. Our results suggest that DIO3OS mainly regulates stemness while moderately affecting the proliferation of HCC cells. This observation is consistent with previous results in other cancers, where DIO3OS was found to regulate metastasis and radiosensitivity, which are closely related to stemness.^[^ ^37^ ^]^ In addition to tumorigenesis and cancer progression, DIO3OS plays a critical role in physiological processes. Recently, DIO3OS was found to preprogram intergenerational brown fat development and obesity resistance in mice via *cis* regulation of DIO3.^[^ ^38^ ^]^ We found that DIO3OS could modulate ZEB1 in a *trans* manner, and that ZEB1 is important in obesity,^[^ ^39^, ^40^ ^]^ suggesting that DIO3OS may also contribute to obesity by regulating ZEB1 in *trans*.

When tumor cells respond to stressful environments caused by malignant proliferation,^[^ ^41^ ^]^ they tend to activate epithelial–mesenchymal transition (EMT) to acquire certain adaptive changes.^[^ ^42^ ^]^ EMT allows cancer cells to detach from the primary tumor and colonize distant sites, leading to extensive proliferation of cancer cells.^[^ ^43^ ^]^ EMT can also confer stemness to tumor cells,^[^ ^44^ ^]^ thus increasing the frequency of tumorigenesis and contributing to increased resistance to therapy and tumor relapse post‐treatment.^[^ ^45^, ^46^ ^]^ Furthermore, the integration of EMT‐inducing transcription factors (TFs) and stemness‐associated signaling into the stemness machinery is believed to modulate the epigenetic circuits of tumor stem cells.^[^ ^4^, ^7^, ^42^, ^47^ ^]^ ZEB1, an EMT‐inducing TF, is a well‐established regulator of tumor stemness^[^ ^48^, ^49^ ^]^ and its expression is tightly controlled. LncRNAs, as master modulators of gene expression, can monitor ZEB1 levels via a variety of mechanisms. LncRNAs have been shown to modulate ZEB1 levels by serving as competing endogenous RNAs for miRNAs, facilitating the transcription of ZEB1^[^ ^50^, ^51^ ^]^ and stabilizing ZEB1 mRNA.^[^ ^52^ ^]^ We found that DIO3OS could regulate the protein, but not mRNA, levels of ZEB1 by modulating the subcellular localization of ZEB1 mRNA. Thus, our study unlocks new insights into the lncRNA‐ZEB1 axis, paving the way for an in‐depth understanding of lncRNA‐based cancer biology. NONO is required for ZEB1 regulation by DIO3OS. Previous studies showed that NONO is almost exclusively observed in the paraspeckles of the nucleus^[^ ^27^, ^53^ ^]^ and is found to play an essential role in mRNA nuclear retention. Surprisingly, we found that NONO could also interact with ZEB1 mRNA, but it mediates the nuclear export of ZEB1 mRNA instead of retaining it in the nucleus, suggesting a novel function of NONO in mRNA biology. It would be of great interest to study the mechanisms underlying the nuclear export function of NONO.

**Figure 8:**
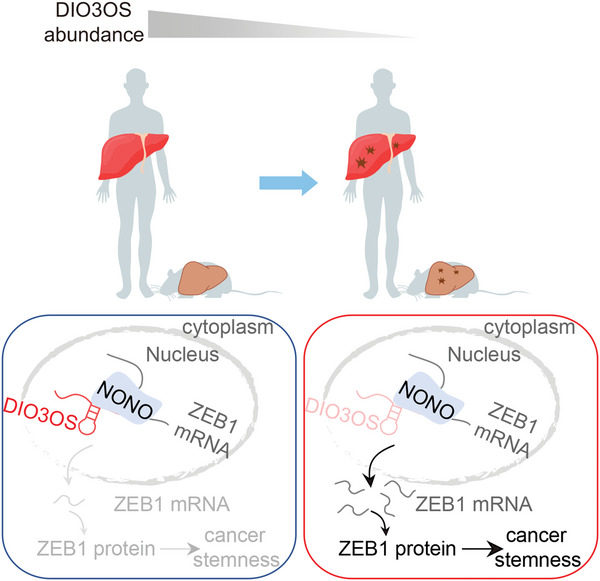
Mechanism of action of DIO3OS in HCC stemness. DIO3OS is a lncRNA conserved across multiple species. DIO3OS blocks the NONO‐mediated ZEB1 mRNA nuclear export, thereby inhibiting liver tumorigenesis, particularly HCC stemness.

In summary, the conserved lncRNA DIO3OS is broadly downregulated in cancers and strongly represses HCC stemness. Mechanistically, DIO3OS bound to NONO and diminished its promoting effect on ZEB1 mRNA nuclear export, thus repressing ZEB1 protein translation and suppressing stemness (**Figure** **8**). Our study revealed a novel mechanism of action of lncRNAs that may provide a better understanding of cancer pathogenesis, thereby offering the possibility for the development of lncRNA‐associated therapeutics.

## 4 Experimental Section

### Clinical Specimens

Human HCC clinical samples were obtained from Third Affiliated Hospital of Sun Yat‐sen University (Guangzhou, China). HCC tumor tissues and matched adjacent non‐tumor tissues were obtained from donors who provided informed consent. Ethical consent was obtained from the Ethics Committee of the Third Affiliated Hospital of Sun Yat‐sen University ([2022]02‐094‐02).

### Cell Culture

The HCC cell lines, Huh7, Hep3B, and SK‐Hep1, were obtained from Prof. Hui Chen (Third Affiliated Hospital of Sun Yat‐sen University, Guangzhou, China). The HCC cell line, HCCLM3, was obtained from Prof. Shi‐Mei Zhuang (Sun Yat‐sen University, Guangzhou, China). HEK293T, Hepa1c1c7, and Hepa1‐6 cells were purchased from the American Type Culture Collection (ATCC). HEK293T, Huh7, SK‐Hep1, and Hepa1‐6 cells were cultured in Dulbecco's modified Eagle's medium (DMEM, Gibco) supplemented with 10% Fetal Bovine Serum (FBS, Gibco) and 1% penicillin/streptomycin (P/S). Hep3B and HCCLM3 cells were cultured in Minimum Essential Medium (MEM, Gibco) supplemented with 10% FBS, 1% non‐essential amino acid solution (Gibco), 1% sodium pyruvate (Gibco), and 1% P/S. Hepa1c1c7 cells were cultured in alpha‐MEM (Gibco) supplemented with 10% FBS and 1% P/S. All cells were maintained at 37 °C with 5% CO_2._

### Plasmids and Stable Cell Lines

For the overexpression plasmids, human DIO3OS was synthesized by Tsingke Biotechnology Co. Ltd. (Beijing, China) and murine Dio3os amplified from mouse liver cDNA were cloned into the pLVX‐puromycin vector, whereas human ZEB1 was inserted into pCDH‐blasticidin for co‐transfection. Specifically, the tRSA sequence amplified from pcDNA3‐tRSA (Addgene) was subcloned between the XbaI and NotI (NEB) sites into the pCDH‐puromycin vector. Human DIO3OS was inserted into the EcoRV site of the pCDH‐tRSA‐puromycin vector. Small guide RNAs (sgRNAs) targeting human DIO3OS, murine Dio3os, human NONO, human ZEB1, and murine Zeb1 were designed online (https://genome.ucsc.edu/). sgRNAs targeting human DIO3OS, mouse Dio3os, or human NONO were annealed and cloned into pLV‐sgRNA‐dCas9‐KRAB‐puromycin vectors. The ZEB1 and NONO sgRNAs were annealed and inserted into the pLenti6.3‐spCas9‐sgRNA‐blasticidin backbone. pT3‐EF1*α*‐c‐Myc‐EGFP (original plasmid from Prof. Xin Chen, University of California San Francisco, California, USA), PX330‐U6‐sgP53‐Cas9 (original plasmid from Addgene) and SB100 (Addgene) plasmids were used to generate primary liver cancer mouse models. Meanwhile, human DIO3OS cDNA and miR30‐based shRNA targeting murine Dio3os (shRNAmir design according to previous articles^[^ ^54^, ^55^ ^]^) were inserted into pT3‐EF1*α*‐c‐Myc‐EGFP vector, respectively. The sgRNA targeting murine Zeb1 was cloned into the PX330‐U6‐sgP53‐Cas9 vector. The corresponding controls for all sgRNAs and shRNAmir did not recognize any sequences in the human and murine genomes. The sequences of the cDNA, sgRNA, and shRNAmir used in this study are listed in Table S2, Supporting Information.

Lentiviruses were produced in HEK293T cells by transfecting the expression plasmids with packaging plasmids (psPAX.2:pMD2.G = 3:1) and a transfection solution (polyethyleneimine; PEI). After incubation for 48 h, the lentivirus media were harvested by centrifuging at 3500 rpm for 5 min and subsequently filtered using 0.45 µm filter. The cells were then infected with lentivirus and selected using puromycin (InvivoGen) or blasticidin (InvivoGen).

### Crystal Violet Staining and Cell Counting Kit‐8 (CCK‐8)

For Crystal violet staining assay, the cells were seeded in seven 12‐well plates at a density of 10^4^ cells/well. At scheduled time points, the cells were fixed with methanol for 10 min and stained with 0.1% crystal violet for 10 min. For CCK‐8 assay, 10^3^ cells were planted in 96‐well plates. Cell viability was measured at the indicated time points using the CCK‐8 reagent (APExBIO), following the manufacturer's instructions.

### Transwell Assay

A total of 5×10^4^–10^5^ cells were diluted in serum‐free medium and planted into the upper chambers, while the lower chambers had 700 µL media containing 20% FBS. After 24 h incubation at 37 °C, the basement membranes of upper chambers were fixed with methanol and stained with 0.1% crystal violet. The upper membranes were not treated in the migration assay, but were coated with 30 µg Matrigel (Corning, DMEM dilution) in the invasion assay. The invading or migrating cells were counted in five random fields per well under a light microscope.

### ALDH Activity Detection

ALDH activity was measured using an ALDEFLUOR kit (STEMCELL) following the manufacturer's instructions.

### Tumorsphere Formation Assay

The cells were harvested using trypsin and washed thrice with PBS. Next, 5×10^3^–10^4^ cells were grown in sphere formation medium (DMEM/F12, Hyclone) supplemented with 1% P/S, 20 ng mL^−1^ recombinant human epidermal growth factor (EGF, Sigma), 10 ng mL^−1^ recombinant human basic fibroblast growth factor (bFGF, R&D Systems), and 1× B27 supplement (Gibco) in six‐well ultra‐low adherent plates. After 7–14 days, the spheres were imaged using a microscope (Leica).

### RNA Extraction and Real‐Time RT‐PCR

Total RNA was purified using TRIzol reagent (Invitrogen). And cDNA was generated using a reverse transcription kit (Vazyme). Real‐time RT‐PCR was performed using the SYBR qPCR Master Mix (Vazyme). All experiments were performed according to the manufacturer's instructions. Relative gene expression levels were calculated by using 2^−ΔΔCt^ method. The primers used are listed in Table S3, Supporting Information.

### Protein Extraction and Western Blotting

Cells were harvested and lysed by RIPA lysis buffer with protease inhibitor (Roche) and phosphatase inhibitor (Roche) for 30 min at 4 °C. Protein was quantified using a BCA kit (KeyGEN) according to the manufacturer's instructions.

For western blotting, 30 µg protein per well was separated by SDS‐PAGE, and transferred to nitrocellulose membrane (Millipore). Then, membranes were blocked with 5% bovine serum albumin (BSA) for 1 h, then incubated with primary antibodies overnight at 4 °C. Horseradish peroxidase‐conjugated secondary antibodies were used to detect primary antibodies. Immunoreactivity was determined using ECL method and imaged using a Bio‐Rad multiple‐function imager. For the cycloheximide (CHX) assay, cells were treated with 50 µg mL^−1^ CHX. At different time points, cells were collected and lysed, and equal amounts of lysate were blotted with antibodies. Protein signals were quantified and normalized. Protein quantification was performed using ImageJ software.

### RNA FISH and Imaging

The cells were seeded onto Millipore slides. Cells were then fixed and permeabilized with fixation solution (methanol: acetic acid = 3:1) for 10 min, followed by washing for 5 min in Wash Buffer A (Biosearch Technologies) supplemented with deionized formamide. Then cells were hybridized with ZEB1 probes in hybridization buffer (Biosearch Technologies) supplemented deionized formamide overnight at 37 °C, and washed with fresh Wash Buffer A for 30 min at 37 °C. Subsequently, cells were counterstained with 5 µg mL^−1^ DAPI for 5 min at 37 °C, followed by washing with Wash Buffer B (Biosearch Technologies) for 5 min. Finally, anti‐fade mounting medium (KeyGEN) was added to the slides and the cells were imaged using a ZEISS confocal laser microscope. The sequences of the anti‐ZEB1 probes with FITC tags are provided in Table S4, Supporting Information.

### tRSA Pull Down

Stable tRSA‐DIO3OS overexpressing and control cells were washed with PBS and crosslinked with fresh 0.75% formaldehyde followed by 1.25 m glycine quenching. The cross‐linked cells were resuspended with RIPA lysis buffer supplemented with protease inhibitor (Roche), phosphatase inhibitor (Roche), and nuclease inhibitor (Accurate Biotechnology, AG), and allowed to sonicate for 20 min with a 5 s on/5 s off cycle at 80% power on a sonicator (SCIENTZ 08‐III) at 4 °C. Insoluble debris was cleared by centrifugation at 14 000 × *g* for 10 min at 4 °C, and supernatant was then incubated with 30 µL streptavidin magnetic beads C1 (Invitrogen) overnight on a rotator at 4 °C. The beads were washed three times with wash buffer (2 × SSC, 0.5% SDS). The beads were divided into two portions (40% for RNA and 60% for protein analysis). Proteins were analyzed using mass spectrometry (MS) or western blotting. To purify RNA, beads were resuspended in 100 µL 1 × DNase buffer supplemented with DNase I (AG) and nuclease inhibitor (AG) followed by incubation at 37 °C for 30 min with 1200 rpm rotation. Beads were digested by the addition of Protease K (AG) at 60 °C for 30 min with 1200 rpm rotation. Next, the MicroElute RNA clean‐up kit (Omega) was used to purify RNA as described in the manufacturer's instructions.

### RNA Immunoprecipitation

For the RIP assay, cells were harvested via tryptic digestion. The cells were then crosslinked and sonicated as described for the tRSA pull‐down assay. Cell lysates were split into two portions, and respectively incubated with anti‐NONO antibodies (Proteintech) or control IgG (Cell Signaling Technology) for 4 h on a rotator at 4 °C. RNA–protein complexes were immunoprecipitated with 30 µL Dynabeads Protein A/G (Thermo Scientific) overnight on a rotator at 4 °C. After incubation with cell lysates, the beads were washed three times with NT2 buffer (50 mm Tris‐HCl (PH 7.4), 1 mm MgCl_2_, 150 mm NaCl_2_, 0.05% NP‐40, and diethyl pyrocarbonate [DEPC] H_2_O). For RNA purification, beads were treated as described for the tRSA pull‐down assay.

### Subcellular Fraction

Subcellular fractionation was performed as previously described.^[^ ^56^, ^57^ ^]^ Cells were collected using trypsin and washed with PBS. Cell pellets were lysed in buffer I (20 mm HEPES, 10 mm KCL, 2 mm MgCl_2_, and 0.5% NP40), and the supernatants were collected for cytoplasmic lysis. Pellets were further lysed in buffer II (0.5 m NaCl, 20 mm HEPES, 10 mm KCL, 2 mm MgCl_2_, and 0.5% NP40) and supernatants were collected for nuclear lysis. Cytoplasmic and nuclear fractions were separated for RNA purification, real‐time RT‐PCR, protein extraction, and western blotting.

### Subcutaneous Injection and Limiting Dilution Assay

Male BALB/c‐nu mice (3.5 weeks) were purchased from Gem Pharmatech (Nanjing, China) and all mice received humane care. For subcutaneous injection, DIO3OS stably overexpressing and control cells or DIO3OS stably depleted and control cells were subcutaneously injected into the left or right flank of the mice. For the limiting dilution assay, different numbers of HCC cells were subcutaneously injected into the left or right flank of mice. Tumor volume was measured every 3–4 days from the second week and was calculated using the following formula: volume = (length× width^2^)/2. When the volume of the tumor reached approximately 1 cm^3^ or the mice developed pathosis, the mice were sacrificed. The frequency of tumor‐initiating cells was estimated using extreme limiting dilution analysis (ELDA) software (http://bioinf.wehi.edu.au/software/elda/). Animal experiments were approved by the Ethics Committee of Guangdong Pharmaceutical University, Guangzhou, China.

### Hydrodynamic Injection for HCC Mice Model

Male C57BL/6J mice (4 weeks old) were purchased from Gem Pharmatech (Nanjing, China) and received humane care. To generate primary liver cancer, 20 µg Myc plasmid with human DIO3OS cDNA or shRNA targeting murine Dio3os (pT3‐EF1*α*‐c‐Myc‐EGFP‐hDIO3OS or pT3‐EF1*α*‐c‐Myc‐EGFP‐mDio3os shRNAmir plasmids), together with 20 µg PX330‐sgP53 plasmids and 1.6 g SB100 plasmids were mixed in 2 mL normal saline. For rescue assay in vivo, 20 µg pT3‐EF1*α*‐c‐Myc‐EGFP‐mDio3os shRNAmir plasmids, together with 20 µg PX330‐sgP53‐sgmZeb1 plasmids and 1.6 g SB100 plasmids were mixed in 2 mL normal saline. The 2 mL plasmids mixture mentioned was injected into mice via the tail vein within 15 s. Detailed methods for the hydrodynamic tail vein injection assay were based on the protocol of Xin Chen Lab (https://pharm.ucsf.edu/xinchen/protocols/hydrodynamic‐tail‐injection) and a previous study.^[^ ^30^ ^]^ Animal experiments were approved by the Ethics Committee of Guangdong Pharmaceutical University, Guangzhou, China.

### Immunohistochemistry

The samples were deparaffinized in xylene, rehydrated in alcohol, and processed as follows. To retrieve antigens, the sections were incubated with the retrieval solution in a steamer for 20 min, followed by treatment with 3% hydrogen peroxide solution for 10 min. And then, sections were incubated overnight in a humid chamber at 4 °C with antibody against Ki‐67 (Abcam) or ZEB1 (Proteintech) followed by secondary antibody (Dako) for 30 min at 37 °C. Immunocomplexes of horseradish peroxidase were visualized using the DAB reaction (Dako), and the sections were counterstained with hematoxylin. Images of the stained sections were captured using a microscope (Nikon).

### Statistical Analysis

All data were obtained from at least three independent experiments. All statistical graphs were shown as mean ± standard deviation (s.d.). Statistical differences were performed using a two‐tailed *t*‐test, one‐way ANOVA test or two‐way ANOVA test in GraphPad Prism 8.0. A *p*‐value less than 0.05 is statistically significant. Sample sizes were selected based on general practices in the field. No statistical methods were used to determine the sample size. The investigators were not blinded to the allocation during the experiments or outcome assessment.

## Conflict of Interest

The authors declare no conflict of interest.

## Author Contributions

Y.‐R.H., L.‐T.D., and Y.‐X.H. contributed equally to this work. Z.‐D.X. conceived the study. Z.‐D.X., Q. Z., and S.‐J.X. supervised the study. Z.‐D.X. and Y.‐R.H. designed the experiments with the help of L.‐T.D., Y.‐X.H. Y.‐R.H. performed most of experiments with the help of L.‐T.D., Y.‐X.H, Q.‐Q.Z., G.L., S.T., W.‐Y.X. The manuscript was written by Z.‐D.X. and Y.‐R.H. with the help of all authors.

## Supporting information

Supporting Information

## Data Availability

The data that support the findings of this study are available in the supplementary material of this article.
