# Supplementary material for: The Conserved LncRNA DIO3OS Restricts Hepatocellular Carcinoma Stemness by Interfering with NONO‐Mediated Nuclear Export of ZEB1 mRNA
Source: Adv Sci (Weinh). 2023 Jun 4;10(23):2301983. doi: 10.1002/advs.202301983 (PMC10427364; doi:10.1002/advs.202301983)
Supplement: Supplementary file 1 — Supporting Information [file ADVS-10-2301983-s001.pdf]

ADVANCED SCIENCE
Open Access

Supporting Information

for *Adv. Sci.*, DOI 10.1002/advs.202301983

The Conserved LncRNA DIO3OS Restricts Hepatocellular Carcinoma Stemness by Interfering with NONO-Mediated Nuclear Export of ZEB1 mRNA

Ya-Rui Hou, Li-Ting Diao, Yan-Xia Hu, Qian-Qian Zhang, Guo Lv, Shuang Tao, Wan-Yi Xu, Shu-Juan Xie, Qi Zhang* and Zhen-Dong Xiao**

Supplementary Figure S1

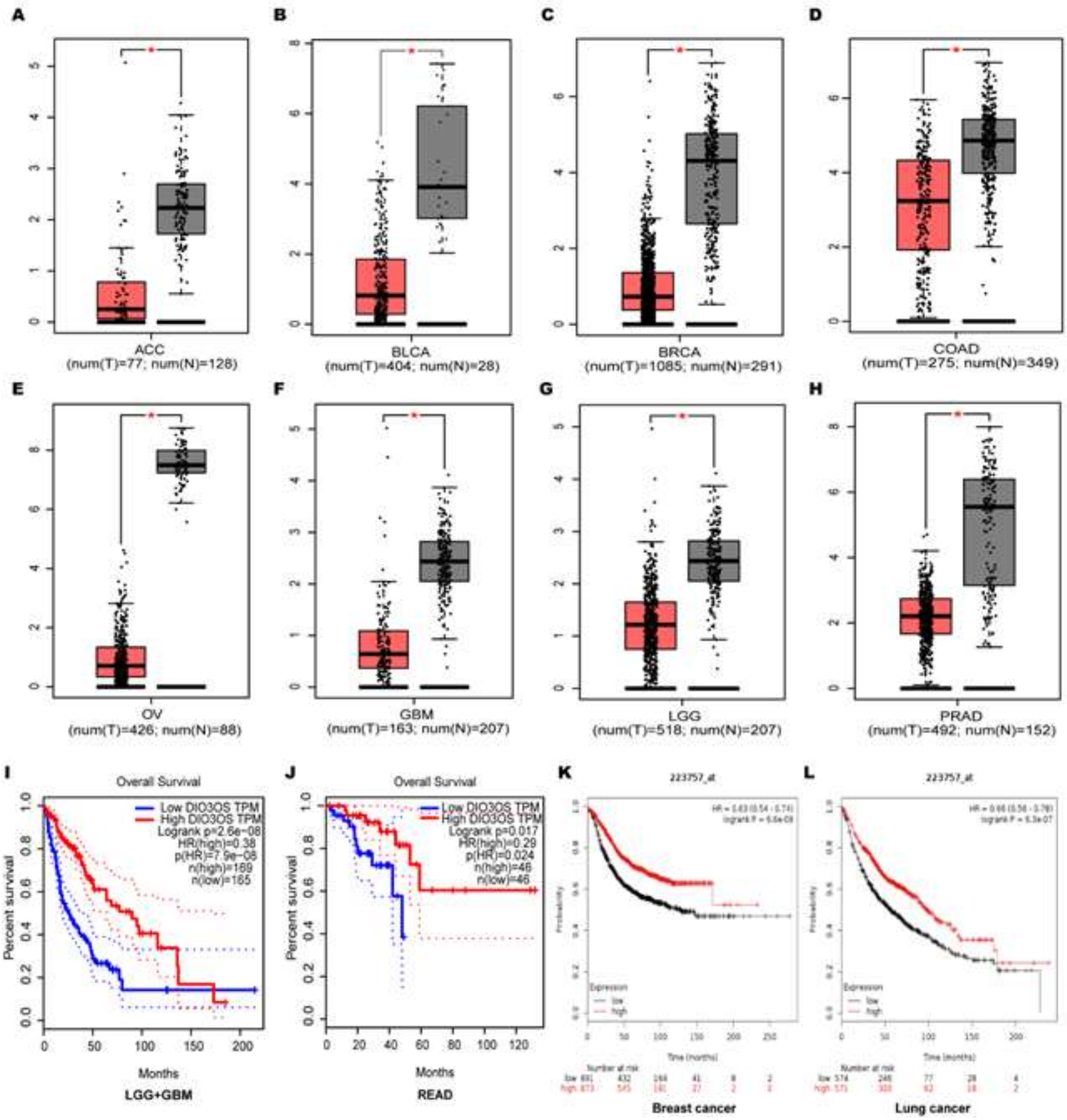

Figure S1. related to Figure 1. DIO3OS is frequently downregulated in many cancers and is associated with patient prognosis.

(A-H) The expression levels of DIO3OS in ACC, BLCA, BRCA, COAD, OV, GBM, LGG and PRAD. Data were from TCGA and GTEx database and analyzed by GEPIA. (I, J) Overall survival analysis performed by GEPIA in brain and rectum adenocarcinoma respectively. GBM and LGG patients were merged. (K, L) Overall survival analysis performed by Kaplan-Meier Plotter

(https://kmplot.com/analysis/) in breast and lung adenocarcinoma respectively. Results were analyzed for probe 223757_at against DIO3OS from microarray data. Abbreviation: ACC, adrenocortical carcinoma; BLCA, bladder urothelial carcinoma; BRCA, breast invasive carcinoma; COAD, colon adenocarcinoma; OV, ovarian serous cystadenocarcinoma; GBM, glioblastoma multiforme; LGG, brain lower grade glioma; PRAD, prostate adenocarcinoma; READ, rectum adenocarcinoma.

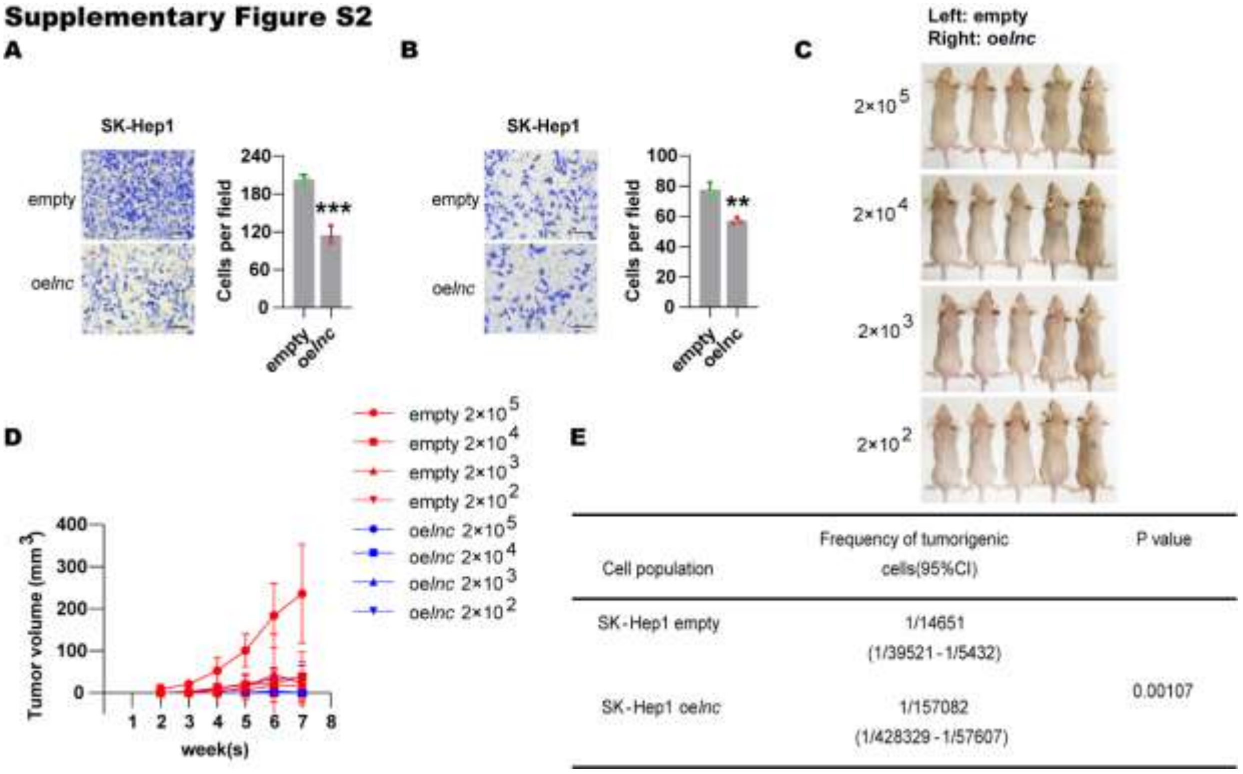

Figure S2. related to Figure 2. Overexpression of DIO3OS suppressed motility and stemness of HCC cells.

(A) Cell invasion was assessed by Transwell invasive assays in SK-Hep1 empty and DIO3OS-overexpressing cells. Representative images were shown, and the numbers of invaded cells were quantified in bar graphs. Scale bars, 100 µm. (B) Cell migration was assessed by Transwell assays without Matrigel in SK-Hep1 empty and DIO3OS-overexpressing cells. Representative images from experiments were shown, and the number of migrated cells were quantified in bar graphs. Scale bars, 100 µm. (C) Different number (2×10^5, 2×10^4, 2×10^3 and 2×10^2) of SK-Hep1 stably overexpressing DIO3OS and empty control cells were subcutaneously injected into the left or the right flanks of mice. Tumor-bearing mice at 7 weeks were shown. n = 5 BALB/C-nu mice for each group. (D) Tumor volumes were measured weekly for two weeks after injection. n = 5 BALB/C-nu mice each group. (E) Tumorigenic cell frequencies of SK-Hep1 empty and DIO3OS overexpressing cells were determined by limiting dilution assay (http://bioinf.wehi.edu.au/software/elda/). Data (Figure S2A, S2B) are presented as mean ± s .d. of three independent experiments and were analyzed by two-tailed Student's *t*-test. ** P <0.01, *** P <0.001.

Supplementary Figure S3

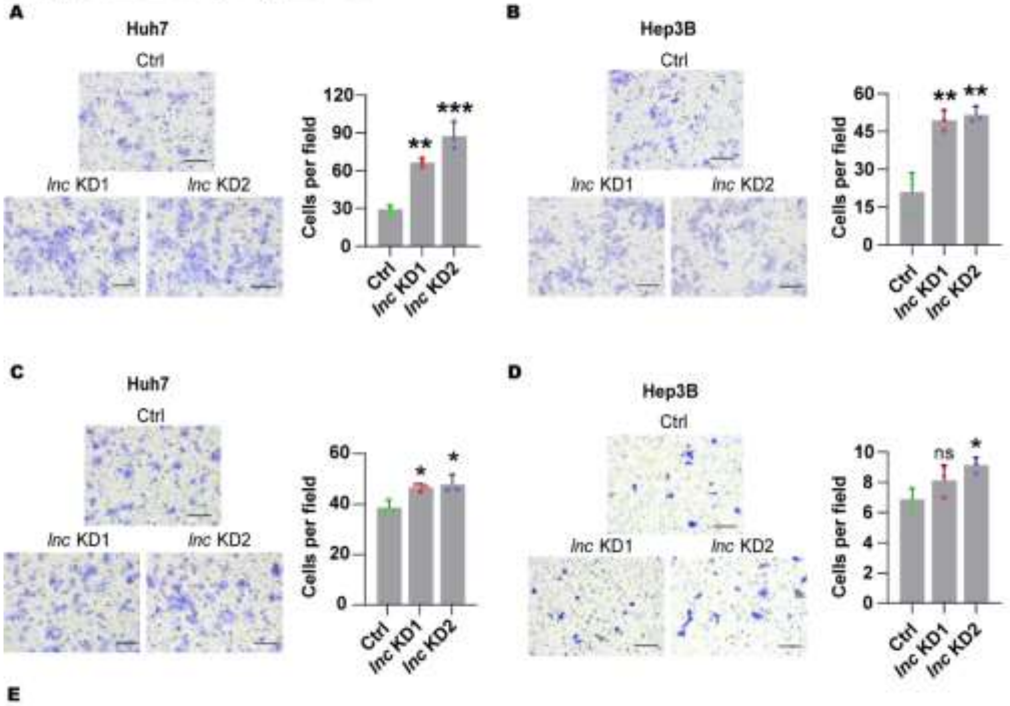

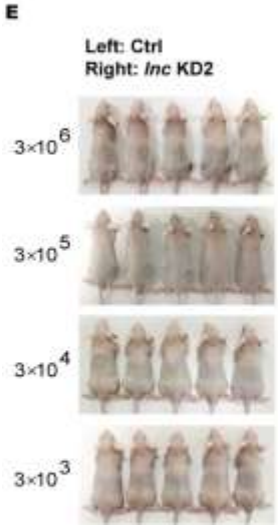

F

Cell population	Frequency of tumorigenic cells(95%CI)	P value
Huh7 Ctrl	1/613493 (1/1879451-1/200258)	0.0166
Huh7 *lnc* KD2	1/91170 (1/254507-1/32659)	

Figure S3. related to Figure 3. DIO3OS depletion promoted invasion and stemness of HCC cells.

(A, B) Cells invasion was measured by Transwell invasive assays in stably expressing control or DIO3OS knocked down Huh7 (A) or Hep3B (B) cells. Representative images from experiments were shown, the numbers of invaded cells were quantified and shown in bar graphs. Scale bars,

100 μm. (C, D) Cells migration was measured by Transwell migrative assays in Huh7 (C) or Hep3B (D) stably DIO3OS depleted cells, respectively. Representative images from experiments were shown, the numbers of migrated cells were quantified and shown in bar graphs. Scale bars, 100 μm. (E) Different number (3×10^6, 3×10^5, 3×10^4 and 3×10^3) of Huh7 stably expressing control or DIO3OS knocked down were subcutaneously injected into the left or the right flanks of mice. Tumor-bearing mice at 46 days were shown. n = 5 BALB/C-nu mice for each group. (F) Tumorigenic cell frequencies of Huh7 stably expressing control or DIO3OS knocked down cells were determined by limiting dilution assay. Data (Figure S3A-D) are shown as mean ± s.d. of three independent experiments and were analyzed by one-way ANOVA with Tukey's multiple comparisons test. * $P < 0.05$, ** $P < 0.01$, *** $P < 0.001$ and ns, not significant.

Supplementary Figure S4

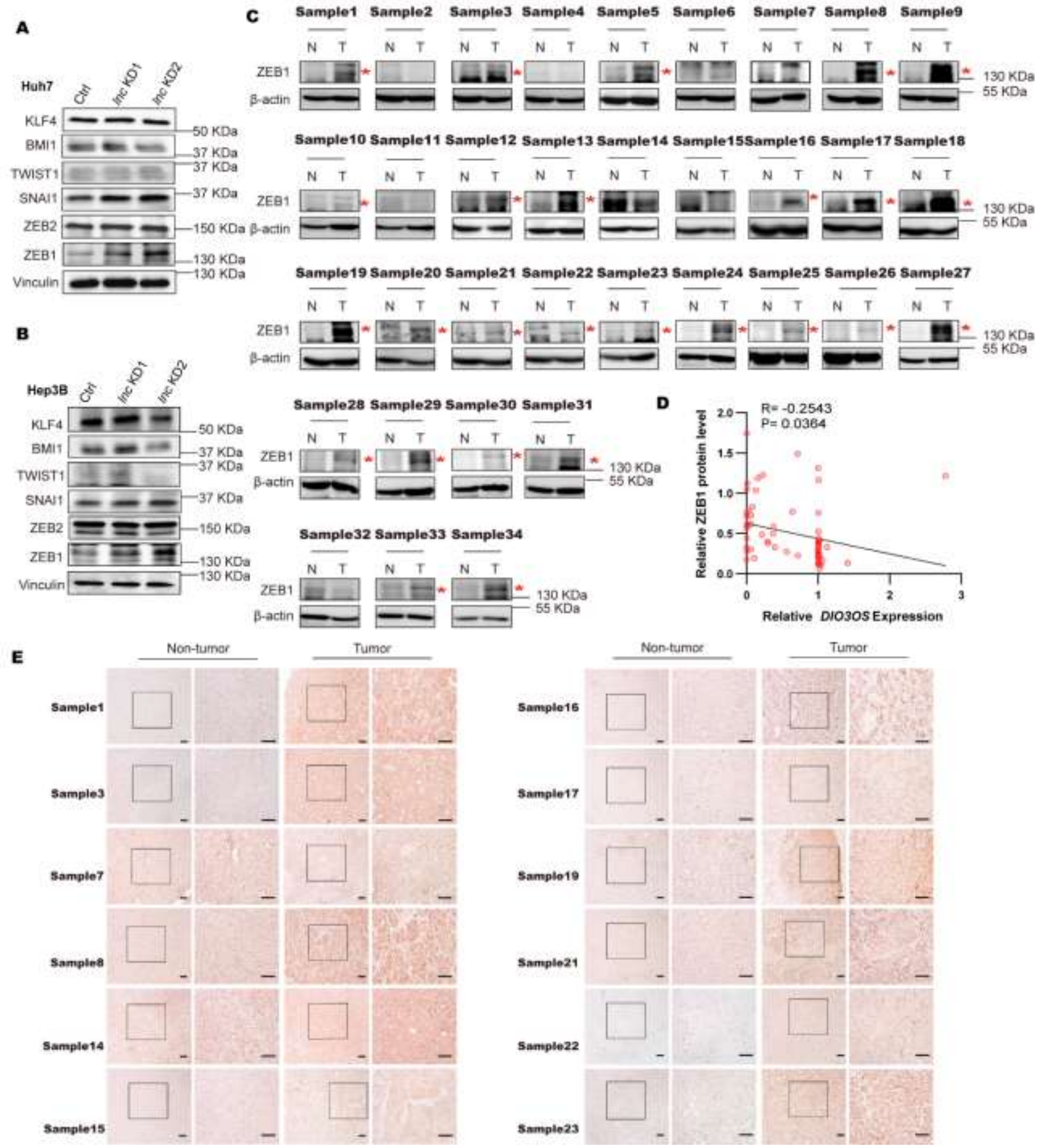

Figure S4. related to Figure 4. ZEB1 was regulated by DIO3OS in HCC.

(A-B) Protein levels of KLF4, BMI1, TWIST1, SNAL1, ZEB2 and ZEB1 were examined in DIO3OS-depleted HCC cells by western blotting, vinculin was used as loading control. (C)

Protein level of ZEB1 was measured in 34 HCC specimens (T = tumor, N = matched adjacent nontumor) by western blotting with β-actin as loading control. (D) Pearson correlation analysis of lncRNA DIO3OS and ZEB1 protein levels in HCC tumor and their matched non-tumor tissues. (E) ZEB1 expression was detected in tumor tissues and their matched nontumor tissues from 12 patients diagnosed with HCC by immunohistochemistry staining. Scale bars, 100 μm.

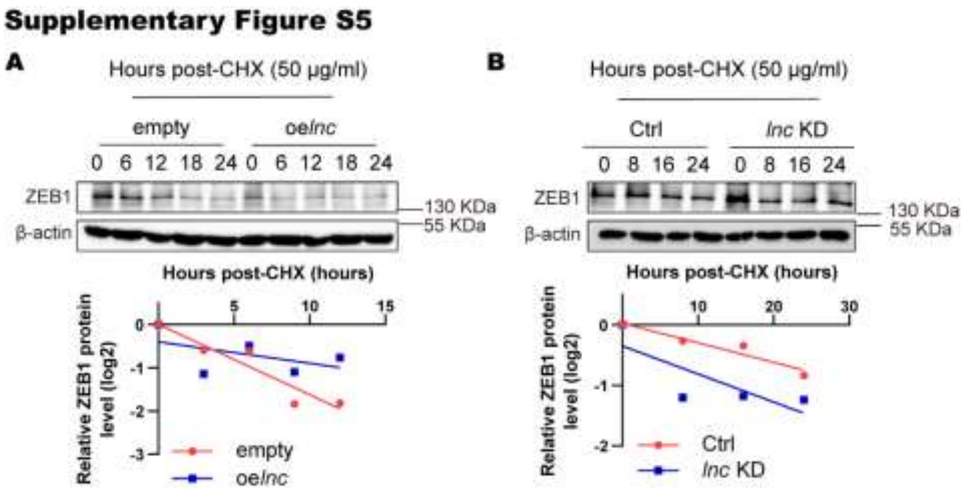

Figure S5. related to Figure 4. DIO3OS stabilizes ZEB1 protein in HCC cells.

(A, B) SK-Hep1 cells stably expressing empty vector or DIO3OS (A) and Huh7 cells stably expressing control sgRNA or DIO3OS targeting sgRNA (B) were treated with cycloheximide (CHX). Cells were collected at different time points and equal amounts of cell lysates were blotted with indicated antibodies. ZEB1 signals (normalized to β-actin) were quantified and plotted.

Supplementary Figure S6

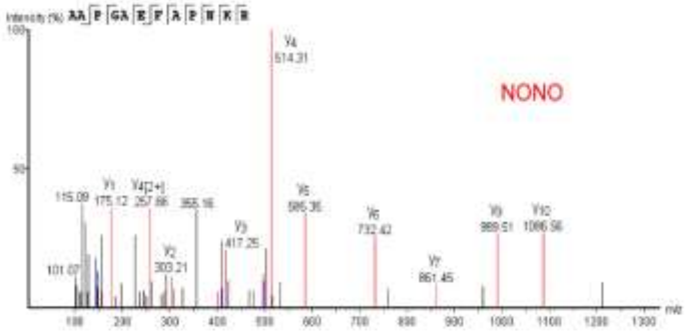

Figure S6. related to Figure 5. Special peptide from NONO was detected by MS.

Supplementary Figure S7

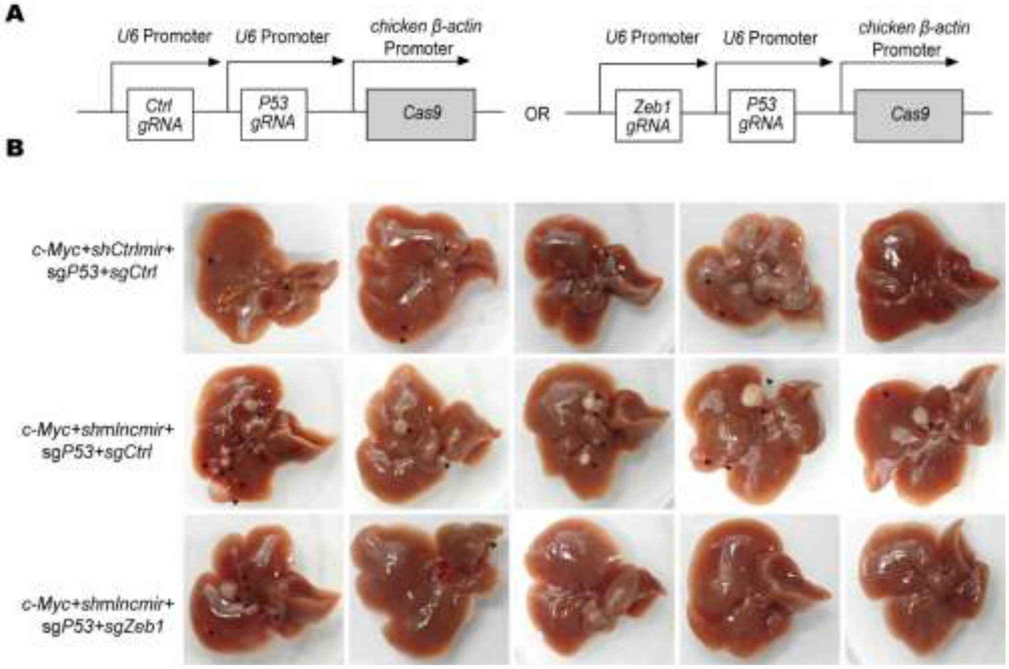

Figure S7. related to Figure 7. DIO3OS played a suppressor role on murine hepatic tumorigenesis by repressing ZEB1 protein.

(A) Diagram of double sgRNA plasmid construction for p53 and Zeb1 depletion. (B) Mice were injected with control group plasmids (*c-Myc*+sh*Ctrlmir*+ sg*P53*+sg*Ctrl*), Dio3os knocked down group plasmids (*c-Myc*+ sh*mlncmir*+sg*P53*+sg*Ctrl*) and Dio3os knocked down + Zeb1 knocked out group plasmids (*c-Myc*+ sh*mlncmir*+sg*P53*+sg*Zeb1*), respectively. Mice were then sacrificed when

the mice were in physiological distress, n = 5 C57BL/6 mice each group. Macroscopic graphs of livers were shown.

Supplemental table S1. MS for tRSA pull down. FC threshold ≥ 3, #peptide ≥ 3.

#Spec tRSA-DIO3OS	#Spec tRSA	FC (tRSA-DIO3OS/tRSA)	Description
10	1	10	Heterogeneous nuclear ribonucleoprotein U OS=Homo sapiens OX=9606 GN=HNRNPU PE=1 SV=6
7	1	7	Heterochromatin protein 1-binding protein 3 OS=Homo sapiens OX=9606 GN=HP1BP3 PE=1 SV=1
7	1	7	Heterogeneous nuclear ribonucleoprotein F OS=Homo sapiens OX=9606 GN=HNRNPF PE=1 SV=3
7	1	7	Core histone macro-H2A.1 OS=Homo sapiens OX=9606 GN=MACROH2A1 PE=1 SV=4
6	1	6	60S ribosomal protein L13 OS=Homo sapiens OX=9606 GN=RPL13 PE=1 SV=4
6	1	6	Heterogeneous nuclear ribonucleoproteins A2/B1 OS=Homo sapiens OX=9606 GN=HNRNPA2B1 PE=1 SV=2
6	1	6	Nucleophosmin OS=Homo sapiens OX=9606 GN=NPM1 PE=1 SV=2
11	2	5.5	Heterogeneous nuclear ribonucleoprotein H OS=Homo sapiens OX=9606 GN=HNRNPH1 PE=1 SV=4
5	1	5	Non-POU domain-containing octamer-binding protein OS=Homo sapiens OX=9606 GN=NONO PE=1 SV=4
5	1	5	Nucleolin OS=Homo sapiens OX=9606 GN=NCL PE=1 SV=3
5	1	5	Elongation factor 1-alpha 1 OS=Homo sapiens OX=9606 GN=EEF1A1 PE=1 SV=1
5	1	5	Putative elongation factor 1-alpha-like 3 OS=Homo sapiens OX=9606 GN=EEF1A1P5 PE=5 SV=1
9	2	4.5	Lamin-B1 OS=Homo sapiens OX=9606 GN=LMNB1 PE=1 SV=2
13	3	4.333333333	Annexin A2 OS=Homo sapiens OX=9606 GN=ANXA2 PE=1 SV=2
16	4	4	Pyruvate carboxylase mitochondrial OS=Homo sapiens OX=9606 GN=PC PE=1 SV=2
4	1	4	Tubulin alpha-1C chain OS=Homo sapiens OX=9606 GN=TUBA1C PE=1 SV=1
4	1	4	Tubulin alpha-1A chain OS=Homo sapiens OX=9606 GN=TUBA1A PE=1 SV=1
4	1	4	Tubulin alpha-1B chain OS=Homo sapiens OX=9606 GN=TUBA1B PE=1 SV=1
4	1	4	40S ribosomal protein S25 OS=Homo sapiens OX=9606 GN=RPS25 PE=1 SV=1
11	3	3.666666667	Heterogeneous nuclear ribonucleoprotein K OS=Homo sapiens OX=9606 GN=HNRNPK PE=1 SV=1
18	5	3.6	Vimentin OS=Homo sapiens OX=9606 GN=VIM PE=1 SV=4
6	2	3	60S ribosomal protein L39 OS=Homo sapiens OX=9606 GN=RPL39 PE=1 SV=2
6	2	3	Putative 60S ribosomal protein L39-like 5 OS=Homo sapiens OX=9606 GN=RPL39P5 PE=5 SV=2
8	0	#DIV/0!	60S ribosomal protein L26 OS=Homo sapiens OX=9606 GN=RPL26 PE=1 SV=1
6	0	#DIV/0!	RNA-binding motif protein X chromosome OS=Homo sapiens OX=9606 GN=RBMX PE=1 SV=3
5	0	#DIV/0!	Poly [ADP-ribose] polymerase 1 OS=Homo sapiens OX=9606 GN=PARP1 PE=1 SV=4
5	0	#DIV/0!	Heterogeneous nuclear ribonucleoprotein A1 OS=Homo sapiens OX=9606 GN=HNRNPA1 PE=1 SV=5

4	0	#DIV/0!	Probable ATP-dependent RNA helicase DDX5 OS=Homo sapiens OX=9606 GN=DDX5 PE=1 SV=1
4	0	#DIV/0!	60S ribosomal protein L35 OS=Homo sapiens OX=9606 GN=RPL35 PE=1 SV=2
3	0	#DIV/0!	DNA topoisomerase 1 OS=Homo sapiens OX=9606 GN=TOP1 PE=1 SV=2
3	0	#DIV/0!	60S ribosomal protein L4 OS=Homo sapiens OX=9606 GN=RPL4 PE=1 SV=5
3	0	#DIV/0!	Heterogeneous nuclear ribonucleoprotein D-like OS=Homo sapiens OX=9606 GN=HNRNPDL PE=1 SV=3
3	0	#DIV/0!	Probable ATP-dependent RNA helicase DDX17 OS=Homo sapiens OX=9606 GN=DDX17 PE=1 SV=2
3	0	#DIV/0!	40S ribosomal protein S23 OS=Homo sapiens OX=9606 GN=RPS23 PE=1 SV=3
3	0	#DIV/0!	Protein DEK OS=Homo sapiens OX=9606 GN=DEK PE=1 SV=1
3	0	#DIV/0!	60S ribosomal protein L34 OS=Homo sapiens OX=9606 GN=RPL34 PE=1 SV=3
3	0	#DIV/0!	Moesin OS=Homo sapiens OX=9606 GN=MSN PE=1 SV=3
3	0	#DIV/0!	60S ribosomal protein L6 OS=Homo sapiens OX=9606 GN=RPL6 PE=1 SV=3
3	0	#DIV/0!	60S ribosomal protein L28 OS=Homo sapiens OX=9606 GN=RPL28 PE=1 SV=3
3	0	#DIV/0!	Heterogeneous nuclear ribonucleoprotein A3 OS=Homo sapiens OX=9606 GN=HNRNPA3 PE=1 SV=2
3	0	#DIV/0!	Serine/arginine-rich splicing factor 3 OS=Homo sapiens OX=9606 GN=SRSF3 PE=1 SV=1
3	0	#DIV/0!	Alpha-enolase OS=Homo sapiens OX=9606 GN=ENO1 PE=1 SV=2
3	0	#DIV/0!	40S ribosomal protein S3a OS=Homo sapiens OX=9606 GN=RPS3A PE=1 SV=2
3	0	#DIV/0!	60S ribosomal protein L37a OS=Homo sapiens OX=9606 GN=RPL37A PE=1 SV=2
3	0	#DIV/0!	GTP-binding nuclear protein Ran OS=Homo sapiens OX=9606 GN=RAN PE=1 SV=3

Supplemental table S2. Sequences of cDNA, sgRNA and shRNA.

Gene Name	Sequence (5'-3')
Murine Dio3os	GAGACTGGAGCGCCCGAACTGAGCCACCTTCGCGAACTCGGAGCGCGGCGGCCCGACTCAACCGCGGAGGCGCCCGGACGCGTAAAGTCAAGGCCTCAACCCGAAGGCAGCGCCTAAGAATCGCA<br>CTCCCTAGAATGCTCCAGCACTCACAGGGGCCTTCTCTGGGGTAACGGGACCCCAGCCGGATACTACAGCTCCAGCTGCTTGCGTCCCATGCATCGCCCTCTCGGGAGAGACCAGCTACCTAACTTTCT<br>CTACCAGAGACCGCGCTGGCGTCTCGGGAGGGATGCTCTGCCCAGGGAGTCCCTCGGTCGCGGGACCTGTGGCCGCCAGAGCAGCTGGATCTGGAGTGTGCTGCCTGTGAACAGCCTGTGAAAGTGG<br>CTCTTCTCCCTCTGGGTCAGAGGATGTCTCTGCTCTGGGGGCAAGGGAGTAAACATCAGCACTTCCCACCTGAAGGAAGCCATCAGACTCCTTTGCTGTTTCTAGCACTCTTTGTAAGGTCTTTCAGGA<br>GGACTTCAGAAATCTCCCTGAACGTGCCTGGCTGGGGGTTCCACTCATGGTTCTATCCCTAAGCAGGGCTCCTCTGAGCTTACAGATCAACCCCCCCCCACTCTCTTTCAGACTTAGGTGGTAAAGTG<br>AGAAGAGACTCTGGCTGGAGAGTCTGAGAGACAGATTGGGAGCTTCTGGGCGCTGTCTTGGGTAGAGCAAGAGATAGTTCTCTTCAGCCCAAGATCCCCTCATCTCTGGGAGATACCAGTCTAGGGCT<br>TGTGTCCTGCTTCTTCCTATAAGGACCTGGGACAGGGATATGAGGCCGATCCTCCCCTTCTCGCATTATGTGTGTAGGTGCATATGCCATGTGGCAGCCATAGTCTGGGACAGATAGAAGTCCTTCTGGTC<br>CTGCTCTATGGGACCAGTCACCCCTGAACATCACCCCTTTCTGATATGGGACACTTGGGAAACCCTGCCATCCACATTTCTGGACAGGGGCGGGTGTTGTGTGGGAAATAGGCTACACCCCACACCTCA<br>GGATTCCAGAGCCTTGGATGAAGAACCCAGGCCTTCGACTTCCCTGGCATCCTGTACAGGGGAGCCCACTTTCTGCCTGGACTCTTCTTGAAGGAGCACACTCCCTCTTCCTGGCCCTTCTTCCTCAGT<br>GGCCAGCAGCCTGTTCAGCAGATGCATTTCTGTGTTTGCCCAGCAGACCTCCCAGGCTGGCCCAGGCGGGATCCCTTGGAGAGACTTCTGGGAGGGGCCCAATAAAGCTTTGTGCAATGAGAAA
Human DIO3OS	CCGCTTGCCTCGCCCAGCGCAGCCCCGGCCGCTGGGCGCACCCGTCCCGTTCGTCCCCGGACGTTGCTCTCTACCCCGGGAACGTCGAGACTGGAGCGCCCGAACTGAGCCACCTTCGCGGACCCCG<br>AGAGCGGCGGCGCGACTCACCGCGGAGGCGCCCGGACGCGTCAAGCCCAGCCCAATAGGAAGCACCTGATCCACACTCTTTCTAGCCCCAGGGATGCTCAGGAAAGCAGCCTTTCTTTAGGGCTGTC<br>ATCTGTTCTGGGGATCTGTTGCTGTTTCTTGGGCCCACAGAATACACTCCACACCTCGGGACTCCATAATATTGGACAAGGCTGGAAGGCCCATTTGGCTCCTGCAGCCTCTTATGCAGGTGAGGACAG<br>ACCCAAGGTCCAGGCAATGCTGGCCTTTGGAACACAGAACCTGGCTCACTGACTCCCACTCCAGTTGTCTTTTCCCTCTGCCTCTGGAGCAACCCTCTCTCCTCCAGAGCAACCCCTGTCCCTCCTTC<br>CTACCTCTCTCTAGGGCTGCACCCCCAGCTCACCTTAGACCTGGCCCTTCCTACCTGTTGCCCCTACTGTCTTGTCCCATCCCAGTGGTCAGGAGGGAGAGTACTGGCCAAAGGCCCAGCTCCACCTGT<br>GACTTAGGGGAGTGCCAGGCATCACCCAGAGGTCCCGGCCCTCGGGGGCCTGGCAGGCTCTGCTGTGGGGGAAGCAGAGTTAGAACTGGGGCTGCTTCTCCTGTCCCCCAGCCTGGCTGGGCCTCCC<br>CCACCTCCCTTTCTGCCTGCCCTCTTACAACTCCAGCAGGGGGCGGGCAGGGGAAAGAAAAGGAGGGACAAGTTGGATGGGGTCCATTTGGTGTCCAGGAACAACTTGGAGAGAGGCGGGGAGGGG<br>ACAGGCAGCAGGGCCCTGGCAGAGCCAGGGGAGGGCTGGCCTTTGGATGGCCTTCTGATCCCAAGCCCATCCTCCTGGGGCAGTCTTCCCTTGAAAGGGAGGGTTTGGTGAACCCTGGTGCGGACCC<br>CCCCGCCGGACCTCACTGCCCCAGTGCGGGGCGTGGTAGGCGGGTTTGCAATGGGTACAGCCTCTCTCTCCATTCCAGTAGAGCAGCTCCCAGGGCCCAGAGAGGCTGTCACTGAGACTTCCCTCTGG<br>GGTGGGGTGGGTTCTGGTGGCCCTGTGTTCCCCTGCAGGAGGACCTCCTGAGGCTTCGGGCAGGAGCTGGTGCACACCTGGAGGAGAGTGCTGGTGAATGGCAGGTGTGTGCCTCAGTGTCCTTGCT<br>GCCAGGGTGTCAGCCCTGCTTCACCACTGGCCGTTTGTCTCTGTCCCCTCCACTGGCCTCTTGAAGCACAGAGACCCTCCGGGTACTTTCCTTCCTCTGGGCTTCCACTCATGCTATTCCCTGCTCCCTT<br>TTTCTACCTGGAGACCCCCATTCCTCCTCCTCCTCTGCTGCCCTCACCTCCACCTGGGCCCTCCCAGCTCCCGGGAGATGGCTGAACCCCACTCCCCACCCGCTAGTCATCCTCTTCAGCGGTCCTCCCA<br>GAGCTGTCCTCAGCATGGCAGCCTCCACAGCCCCTGGTGTCACCCCCGGTTTCGTGTCTGCCTCGGACTCCAGACCTGCCCCTCCCAGGCTGGCCCAGATGGATCCTCTACAGTGACTAACCCAAACCA<br>GCACCAGGCAGGGACCCAATAAACCTTTGTGAATGGAGT

sgRNA	Forward sequence (5'-3')	Reverse sequence (5'-3')
nontarget gRNA	caccGCGGGCAGAACGACCCTGAC	aaacGTCAGGGTCGTTCTGCCCGC
Human DIO3OS gRNA-1	caccAGGAAGCACCTGATCCACAC	aaacGTGTGGATCAGGTGCTTCCT
Human DIO3OS gRNA-2	caccCGGACGTTGCTCTCTACCCC	aaacGGGGTAGAGAGCAACGTCCG
Murine Dio3os gRNA-1	caccAGGTCAAGGCCTCAACCCGA	aaacTCGGGTTGAGGCCTTGACCT
Murine Dio3os gRNA-2	caccTGACCTGAGGGGTCATTGTG	aaacCACAATGACCCCTCAGGTCA
Human NONO gRNA-1	caccAGGAGATACCAGTCGGTAGA	aaacTCTACCGACTGGTATCTCCT
Human NONO gRNA-2	caccTACTCCGAGGAGATACCAGT	aaacACTGGTATCTCCTCGGAGTA
Human ZEB1 gRNA	caccGGCGCAATAACGGTGAGTGG	aaacCCACTCACCGTTATTGCGCC
Murine ZEB1 gRNA	caccGCACAAGCGAGAGGATCATGG	aaacCCATGATCCTCTCGCTTGTGC

shRNA	Forward sequence (5'-3')	Reverse sequence (5'-3')
control	AGCGCCTAAGGTTAAGTCGCCCTCGTAGTGAAG	GGCACCTAAGGTTAAGTCGCCCTCGTACATCTG
shRNAmir	CCACAGATGTACGAGGGCGACTTAACCTTAGG	TGGCTTCACTACGAGGGCGACTTAACCTTAGG
Murine Dio3os	AGCGAGAATCGCACTCCCTAGAATGTAGTGAAG	GGCAAGAATCGCACTCCCTAGAATGTACATCTG
shRNAmir	CCACAGATGTACATTCTAGGGAGTGCGATTCT	TGGCTTCACTACATTCTAGGGAGTGCGATTCT

Supplemental table S3. Primer sequences for real-time RT-PCR.

gene name	Forward Sequence (5'-3')	Reverse Sequence (5'-3')
Human GAPDH	GTCTCCTCTGACTTCAACAGCG	ACCACCCTGTTGCTGTAGCCAA
Murine GAPDH	CATCACTGCCACCCAGAAGACTG	ATGCCAGTGAGCTTCCCGTTCAG
Human DIO3OS	AGGAAGCACCTGATCCACAC	CCTTCCAGCCTTGTCCAATA
Murine Dio3os	CGCACTCCCTAGAATGCTCC	TAGGTAGCTGGTCTCTCCCG
Human NONO	CATCAAGGAGGCTCGTGAGAAG	TGGTTGTGCAGCTCTTCCATCC
Human ZEB1	AGCAGTGAAAGAGAAGGG	GGTCCTCTTCAGGTGCCT
Murine ZEB1	ATTCAGCTACTGTGAGCCCTGC	CATTCTGGTCCTCCACAGTGGA
Human U1	TCCCAGGGCGAGGCTTATCCATT	GAACGCAGTCCCCCACTACCACAAAT
Human NEAT1	GCTGGACCTTTCATGTAACGGG	TGAACTCTGCCGGTACAGGGAA

Supplemental table S4. Probes against human ZEB1 for RNA FISH.

#Probe	Sequence (5'- 3')	Position	GC%
1	TGCAGTTTGTCTTCATCATC	49	40.00%
2	TTTTACTGTACATCCTGCTT	257	35.00%
3	ATGACAGCAGTGTCTTGTTG	346	45.00%
4	AAATGCATCTGGTGTTCCAT	431	40.00%
5	GTAAAGCGTTTATAGCCTCT	481	40.00%
6	GCACAGGGAGCAACTAAAGT	548	50.00%
7	GTTCAAGTTGGGTTCTGTAT	582	40.00%
8	CGTCACATGTCTTTGATCTC	629	45.00%
9	AGCCAGAATGGGAAAAGCGT	777	50.00%
10	ACTGCTTATGTGTGAGCTAT	800	40.00%
11	CCTGGTGATGCTGAAAGAGA	895	50.00%
12	AGTTGTTCTTGAAGGGGTTT	955	40.00%
13	TCATAATCCACAGGTTCAGT	994	40.00%
14	TTGATTCCTGAAGCAACCAC	1027	45.00%
15	CAGTGAAAACCCCATTTTGT	1062	40.00%
16	GAGAACTGGTTGCCTGTAAT	1092	45.00%
17	CTTATGGGAGACACCAAACC	1150	50.00%
18	CGCCACTTTAAGTACATTCT	1190	40.00%
19	CTTGCCTTATTACATTACCA	1215	35.00%
20	ACCTTGTTGTATGGGTGAAG	1289	45.00%
21	GTTCCATCTTGATCAACCAA	1342	40.00%
22	TCAAAGCTTTTGTCCTTCTC	1501	40.00%
23	GACAAGTGCTATCATTCACC	1527	45.00%
24	ATCTCCTGGACAATCATCAC	1553	45.00%
25	GGTTCTTTAAAGGTGGCTGA	1707	45.00%
26	ATTTTTGAGAGCTCTTCTGC	1774	40.00%
27	TTACTACATCCAGTGGTAGG	1809	45.00%
28	CTGGTTCAGGAGAAGATGGT	1881	50.00%
29	GTTCTTGGCAGGGATATTTA	1907	40.00%
30	TTTGCAGATTGAGGCTGATC	1930	45.00%
31	CCACTGGTAAAACTGGGGAG	2007	55.00%
32	AGTAACCCTGTGTATTTCTG	2091	40.00%
33	AATAATTCTCCCTGTTGCTT	2173	35.00%
34	TCCTTTTTTGCGCAAGACAA	2260	40.00%
35	CTGGTTCTGAGTCTGTAACA	2298	45.00%
36	GATATTTATGGGGTTGGCAC	2339	45.00%
37	TAACTGGGCAGTGACTGTAG	2366	50.00%
38	TAGCGCTCTTAAGCATGGAA	2420	45.00%
39	GGGGAATCAGAATCGTTTGC	2451	50.00%
40	CTGGATCACTTTCAAGGGTG	2519	50.00%

41	CTTGTCTTTCATCCTGATTT	2547	35.00%
42	TGATACTCCTTCTGAGCTAG	2570	45.00%
43	AGAGTCATTCTGATCCTCTA	2594	40.00%
44	ACATTCCATTTTCTGTCTTC	2643	35.00%
45	TCTTTTACCTGTGTGTTCAT	2723	35.00%
46	CCTTTTTACAGATTCCACAC	2751	40.00%
47	CTCTTACAGTAGGAGTAGCG	2896	50.00%
48	CTCTTACAGTAGGAGTAGCG	2896	50.00%

Supplemental table S5. Reagents or resources information.

Reagents or resources	Source	Catalog No.
**Antibodies**		
Rabbit anti-GAPDH	Cell Signaling Technology	Cat# 2118
Rabbit anti-LaminA/C	Cell Signaling Technology	Cat# 2032
Mouse anti-Vinculin	Sigma	Cat# V9264
Rabbit anti-ZEB1	Proteintech	Cat# 21544-1-AP
Rabbit anti-ZEB2	Proteintech	Cat# 14026-1-AP
Rabbit anti-TWIST1	Proteintech	Cat# 25465-1-AP
Rabbit anti-TWIST2	Proteintech	Cat# 11752-1-AP
Rabbit anti-SNAI1	Proteintech	Cat# 13099-1-AP
Rabbit anti-SNAI2	Proteintech	Cat# 12129-1-AP
Rabbit anti-ALDH1A1	Proteintech	Cat# 15910-1-AP
Rabbit anti-NONO	Proteintech	Cat# 11058-1-AP
Rabbit anti-SFPQ	Proteintech	Cat# 15585-1-AP
Rabbit anti-BMI1	ABclonal	Cat# A0211
Rabbit anti-KLF4	ABclonal	Cat# A6640
Rabbit anti-OCT4	ABclonal	Cat# A7920
Rabbit anti- β-actin	Bioworlde	Cat# I102
HRP-conjugated anti-rabbit IgG antibody	Cell Signaling Technology	Cat# 7074
HRP-conjugated anti-mouse IgG antibody	Cell Signaling Technology	Cat# 7076
Rabbit anti-Ki-67	Abcam	Cat# ab15580
**Bacterial Strain**		
Stb13	KangTi Life	Cat# KTSM110L
**Chemicals**		
Opti-MEM	GIBCO	Cat# 31985062
Streptavidin magnetic beads C1	Invitrogen	Cat# 65001
Dynabeads Protein A/G	Thermo Scientific	Cat# 88803
Recombinant human epidermal growth factor	Sigma	Cat# 11376454001
Recombinant human basic fibroblast growth factor	R&D Systems	Cat# 233-FB-025
Matrigel	Corning	Cat# 354234
Recombinant RNase Inhibitor	Accurate Biology	Cat# AG11608
DNase I (RNase Free)		
10X DNase I Buffer	Accurate Biology	Cat# AG12001
Critical Commercial Assays		
Hiscript® III RT SuperMix for qPCR (+gDNA wiper)	Vazyme	Cat# R323-01
ChamQ Universal SYBR qPCR Master Mix	Vazyme	Cat# Q711-02
CCK-8 Kit	APExBIO	Cat# 1018
ALDEFLUOR™ Kit	STEMCELL	Cat# 01700
MicroElute RNA Clean Up Kit	Omega	Cat# R6247-02
**Experimental Models: Cell Lines**		
HEK293	ATCC	Cat# CRL-1573
HCCLM3	From Prof. Shi-Mei Zhuang	N/A
Hepa1c1c7	ATCC	Cat# CRL-2026
Hepa1-6	ATCC	Cat# CRL-1830

SK-Hep1	From Prof. Hui Chen	N/A
Huh7	From Prof. Hui Chen	N/A
Hep3B	From Prof. Hui Chen	N/A
**Experimental Models: Organisms/Strains**		
BALB/c nude mice	Gem Pharmatech	N/A
C57BL/6J mice	Gem Pharmatech	N/A
**Oligonucleotides**		
cDNA, sgRNA, shRNA sequence, see Table S2	This paper	N/A
Primers for real-time RT-PCR, see Table S3	This paper	N/A
Probes for RNA FISH, see Table S4	This paper	N/A
**Recombinant DNA**		
tRSA plasmid	addgene	Cat# 32200
PX330-U6-sgP53-Cas9	addgene	Cat# 42230
SB100	addgene	Cat# 34879
**Software**		
GraphPad Prism 8.0	GraphPad Software	https://www.graphpad.com
Extreme Limiting Dilution Analysis (ELDA) Software	ELDA Software	http://bioinf.wehi.edu.au/software/elda/
